# Supplementary material for: Timescales of Massive Human Entrainment
Source: PLoS One. 2015 Apr 16;10(4):e0122742. doi: 10.1371/journal.pone.0122742 (PMC4400172; doi:10.1371/journal.pone.0122742)
Supplement: S1 Code — The file is a pdf containing the code used to run the analyses, commented for understandability, and the output of the code. (PDF) [file pone.0122742.s001.pdf]

# A demonstration of the data fusion, showing Twitter and dynamic interaction data, overlaid (Debate 1)

As in the initial script, import libraries and specify time alignment parameters so we can fuse Twitter with interactive data.

```
library(lme4)
library(languageR)
library(ggplot2)

token_transcript_mention = c(26*60+11,38*60+44,42*60+3)
transcripts_time_starts = c('Wed Oct  3 21:01:44 2012','Tue Oct 16 21:01:49 2012','Mon Oct 22 21:01:52 
```

As described in the "all data and alignment" markdown, we utilize intervals defined the timecodes.txt file. This allows plotting of rect structures with different alpha levels to be overlaid on top of tweet rate /s.

```
dnum = 1 # debate number

debate = read.table(paste('../data/debate',dnum,'.txt',sep=''),head=TRUE,sep='\t')
colnames(debate) = c('date','total','obama','romney','token')

align_index = which(debate$date==transcripts_time_starts[dnum]) ### let's align the debate twitter data
debate = debate[align_index:nrow(debate),]

timecodes = read.table('../data/timecodes.txt',sep='\t',head=FALSE)
colnames(timecodes) = c('startt','endt','who','speechturn','dnum','interrupt1','interrupt2','topic')
timecodes = timecodes[timecodes$dnum==dnum,]

speakers = c('Romney','Obama','Moderator','Questioner (Debate 2)')
colors = c('red','blue')
```

Here we begin the plot. In rects we plot where speaking is taking place among candidates (colored by party); points reflect tweet rate / s across the time course of the debate. y-axis = tweets / second; x=axis = time (seconds) into debate.

```
#dev.new(width=4.3, height=3)

plot(debate$obama,col=rgb(0,0,1,.5),xlim=c(1,length(debate$obama)),cex=.15,type='p',ann=FALSE,cex.axis=
mtext(side = 1, text = "", line = 1,cex=.75)
mtext(side = 2, text = "", line = 1.25,cex=.75)
points(debate$romney,col=rgb(1,0,0,.5),cex=.15,type='p')
mtext(side = 3, text = "", line = .5,cex=.75)

oboundaries = timecodes[timecodes$who==2,]
rboundaries = timecodes[timecodes$who==1,]
for (i in 1:dim(oboundaries)[1]) {
    rect(oboundaries[i,1],-100,oboundaries[i,2],200,col=rgb(0,0,1,.2),border=FALSE)
}
for (i in 1:dim(rboundaries)[1]) {
    rect(rboundaries[i,1],-100,rboundaries[i,2],200,col=rgb(1,0,0,.2),border=FALSE)
}
```

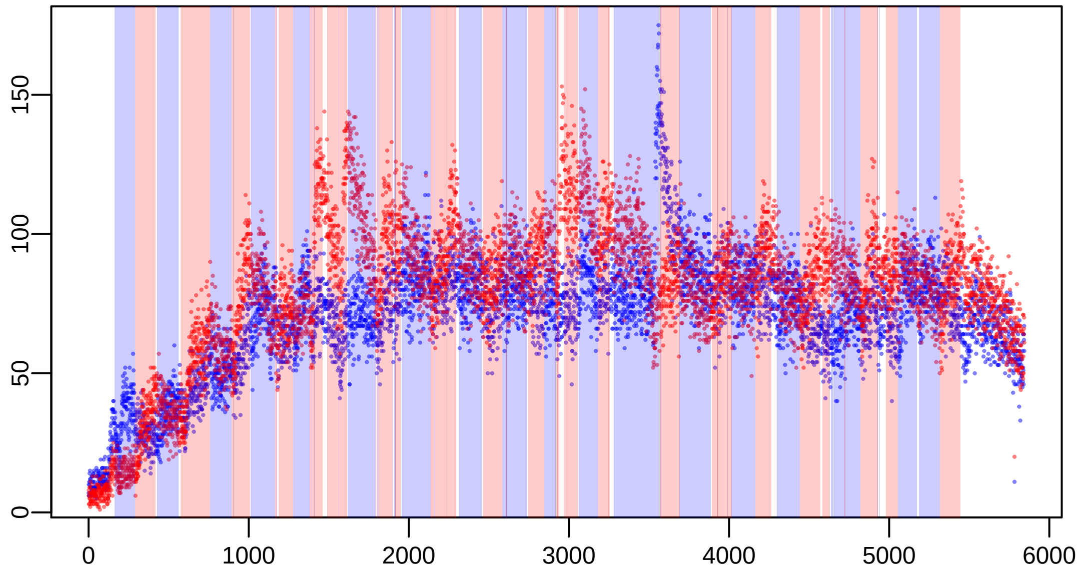


```r
#dev.off()
```

The following is an important set of information regarding the data coding and analysis.

Debate 1 starts in transcripts: 9:01:44.

Debate 2 starts in transcripts: 9:01:49.

Debate 3 starts in transcripts: 9:01:52

Timed using stopwatch + CSPAN clock – at the onset of speech.

From looking into the video / transcript:

big bird: 26:11

binders full of women 38:44

[horses and ]bayonets 42:03

For the time codes:

3-Interlocutor (1=Romney, 2=Obama, 3=Moderator, 4=Questioner in debate2)

6-Interruptions1 (1=interruption, 2=interrupting and taking the ground)

7-Interruptions2 (1=Moderator interrupts Romney, 2=M interrupts O, 3=O interrupts R, 4=R interrupts O, 5=R interrupts M, 6=O interrupts M)

# A demonstration of the data fusion, showing Twitter and dynamic interaction data, overlaid (Debate 2)

As in the initial script, import libraries and specify time alignment parameters so we can fuse Twitter with interactive data.

```
library(lme4)
library(languageR)
library(ggplot2)

token_transcript_mention = c(26*60+11,38*60+44,42*60+3)
transcripts_time_starts = c('Wed Oct  3 21:01:44 2012','Tue Oct 16 21:01:49 2012','Mon Oct 22 21:01:52 
```

As described in the “all data and alignment” markdown, we utilize intervals defined the timecodes.txt file. This allows plotting of rect structures with different alpha levels to be overlaid on top of tweet rate /s.

```
dnum = 2 # debate number

debate = read.table(paste('../data/debate',dnum,'.txt',sep=''),head=TRUE,sep='\t')
colnames(debate) = c('date','total','obama','romney','token')

align_index = which(debate$date==transcripts_time_starts[dnum]) ### let's align the debate twitter data
debate = debate[align_index:nrow(debate),]

timecodes = read.table('../data/timecodes.txt',sep='\t',head=FALSE)
colnames(timecodes) = c('startt','endt','who','speechturn','dnum','interrupt1','interrupt2','topic')
timecodes = timecodes[timecodes$dnum==dnum,]

speakers = c('Romney','Obama','Moderator','Questioner (Debate 2)')
colors = c('red','blue')
```

Here we begin the plot. In rects we plot where speaking is taking place among candidates (colored by party); points reflect tweet rate / s across the time course of the debate. y-axis = tweets / second; x=axis = time (seconds) into debate.

```
#dev.new(width=4.3, height=3)

plot(debate$obama,col=rgb(0,0,1,.5),xlim=c(1,length(debate$obama)),cex=.15,type='p',ann=FALSE,cex.axis=
mtext(side = 1, text = "", line = 1,cex=.75)
mtext(side = 2, text = "", line = 1.25,cex=.75)
points(debate$romney,col=rgb(1,0,0,.5),cex=.15,type='p')
mtext(side = 3, text = "", line = .5,cex=.75)

oboundaries = timecodes[timecodes$who==2,]
rboundaries = timecodes[timecodes$who==1,]
for (i in 1:dim(oboundaries)[1]) {
    rect(oboundaries[i,1],-100,oboundaries[i,2],200,col=rgb(0,0,1,.2),border=FALSE)
}
for (i in 1:dim(rboundaries)[1]) {
    rect(rboundaries[i,1],-100,rboundaries[i,2],200,col=rgb(1,0,0,.2),border=FALSE)
}
```

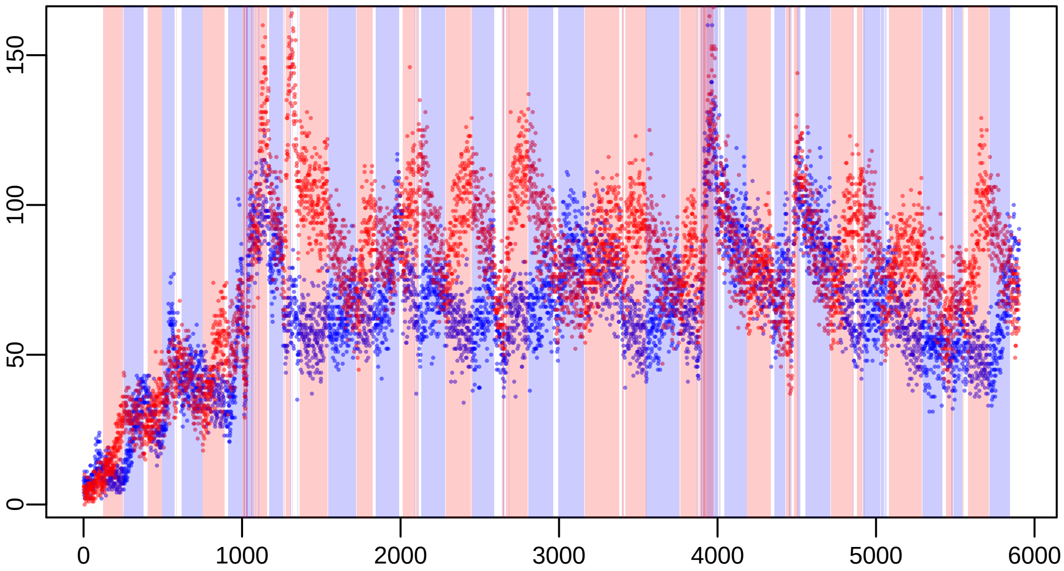


```
#dev.off()
```

The following is an important set of information regarding the data coding and analysis.

Debate 1 starts in transcripts: 9:01:44.

Debate 2 starts in transcripts: 9:01:49.

Debate 3 starts in transcripts: 9:01:52

Timed using stopwatch + CSPAN clock – at the onset of speech.

From looking into the video / transcript:

big bird: 26:11

binders full of women 38:44

[horses and ]bayonets 42:03

For the time codes:

3-Interlocutor (1=Romney, 2=Obama, 3=Moderator, 4=Questioner in debate2)

6-Interruptions1 (1=interruption, 2=interrupting and taking the ground)

7-Interruptions2 (1=Moderator interrupts Romney, 2=M interrupts O, 3=O interrupts R, 4=R interrupts O, 5=R interrupts M, 6=O interrupts M)

# A demonstration of the data fusion, showing Twitter and dynamic interaction data, overlaid (Debate 3)

As in the initial script, import libraries and specify time alignment parameters so we can fuse Twitter with interactive data.

```
library(lme4)
library(languageR)
library(ggplot2)

token_transcript_mention = c(26*60+11,38*60+44,42*60+3)
transcripts_time_starts = c('Wed Oct  3 21:01:44 2012','Tue Oct 16 21:01:49 2012','Mon Oct 22 21:01:52
```

As described in the "all data and alignment" markdown, we utilize intervals defined the timecodes.txt file. This allows plotting of rect structures with different alpha levels to be overlaid on top of tweet rate /s.

```
dnum = 3 # debate number

debate = read.table(paste('../data/debate',dnum,'.txt',sep=''),head=TRUE,sep='\t')
colnames(debate) = c('date','total','obama','romney','token')

align_index = which(debate$date==transcripts_time_starts[dnum]) ### let's align the debate twitter data
debate = debate[align_index:nrow(debate),]

timecodes = read.table('../data/timecodes.txt',sep='\t',head=FALSE)
colnames(timecodes) = c('startt','endt','who','speechturn','dnum','interrupt1','interrupt2','topic')
timecodes = timecodes[timecodes$dnum==dnum,]

speakers = c('Romney','Obama','Moderator','Questioner (Debate 2)')
colors = c('red','blue')
```

Here we begin the plot. In rects we plot where speaking is taking place among candidates (colored by party); points reflect tweet rate / s across the time course of the debate. y-axis = tweets / second; x=axis = time (seconds) into debate.

```
#dev.new(width=4.3, height=3)

plot(debate$obama,col=rgb(0,0,1,.5),xlim=c(1,length(debate$obama)),cex=.15,type='p',ann=FALSE,cex.axis=
mtext(side = 1, text = "", line = 1,cex=.75)
mtext(side = 2, text = "", line = 1.25,cex=.75)
points(debate$romney,col=rgb(1,0,0,.5),cex=.15,type='p')
mtext(side = 3, text = "", line = .5,cex=.75)

oboundaries = timecodes[timecodes$who==2,]
rboundaries = timecodes[timecodes$who==1,]
for (i in 1:dim(oboundaries)[1]) {
    rect(oboundaries[i,1],-100,oboundaries[i,2],200,col=rgb(0,0,1,.2),border=FALSE)
}
for (i in 1:dim(rboundaries)[1]) {
    rect(rboundaries[i,1],-100,rboundaries[i,2],200,col=rgb(1,0,0,.2),border=FALSE)
}
```

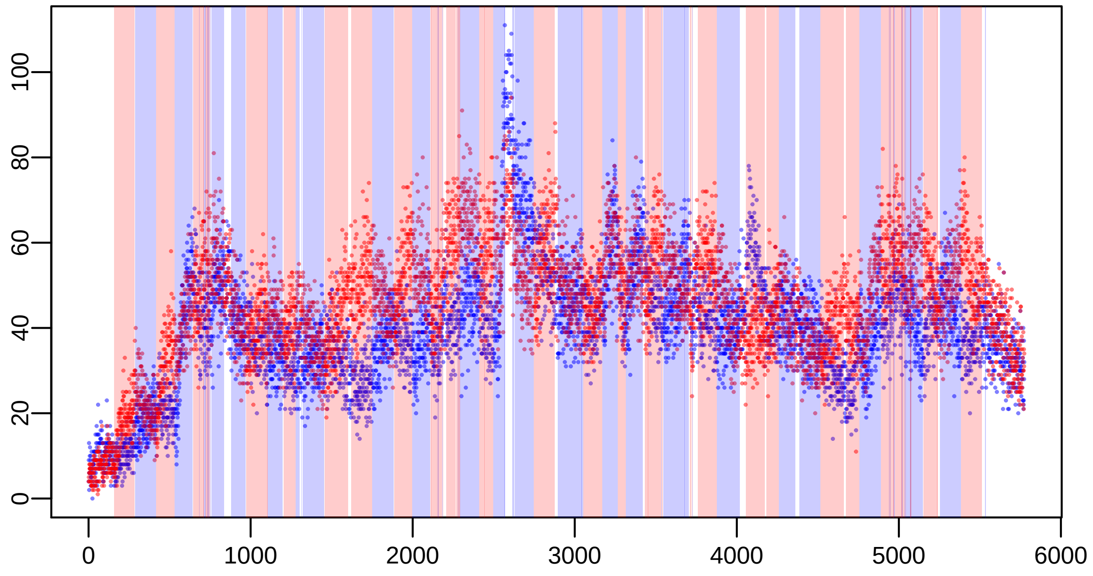


```
#dev.off()
```

The following is an important set of information regarding the data coding and analysis.

Debate 1 starts in transcripts: 9:01:44.

Debate 2 starts in transcripts: 9:01:49.

Debate 3 starts in transcripts: 9:01:52

Timed using stopwatch + CSPAN clock – at the onset of speech.

From looking into the video / transcript:

big bird: 26:11

binders full of women 38:44

[horses and ]bayonets 42:03

For the time codes:

3-Interlocutor (1=Romney, 2=Obama, 3=Moderator, 4=Questioner in debate2)

6-Interruptions1 (1=interruption, 2=interrupting and taking the ground)

7-Interruptions2 (1=Moderator interrupts Romney, 2=M interrupts O, 3=O interrupts R, 4=R interrupts O, 5=R interrupts M, 6=O interrupts M)

# A breakdown of all data and the data fusion process, along with initial speaker analysis (Debate 1)

First, we load in required libraries and specify variables that were determined in order to time-align the Twitter and CSPAN feeds. CSPAN times were determined by the by-the-second debate initiation using real time. Token mention is the time, in seconds, at which the salient moment was introduced in the debate (see the paper for more details).

```
library(lme4)
library(languageR)
library(ggplot2)
library(MuMIn)

token_transcript_mention = c(26*60+11,38*60+44,42*60+3) # time when tokens (salient events) mentioned
transcripts_time_starts = c('Wed Oct  3 21:01:44 2012','Tue Oct 16 21:01:49 2012','Mon Oct 22 21:01:52 2
```

Next, we determine which debate we are analyzing, load in the relevant files, and time-align the Twitter and conversation dynamics. "nort" means that the Twitter data reflects tweets without any retweets.

```
dnum = 1 # debate number

debate = read.table(paste('../data/debate',dnum,'.nort.txt',sep=''),head=TRUE,sep='\t') # load in debat
colnames(debate) = c('date','total','obama','romney','token')

### let's align the debate twitter data
align_index = which(debate$date==transcripts_time_starts[dnum]) # get row number where debate time star
debate = debate[align_index:nrow(debate),] # start debate data from second of debate onset

timecodes = read.table('../data/timecodes.txt',sep='\t',head=FALSE) # time codes from aarhus
colnames(timecodes) = c('startt','endt','who','speechturn','dnum','interrupt1','interrupt2','topic')
timecodes = timecodes[timecodes$dnum==dnum,] # take only the current debate

speakers = c('Romney','Obama','Moderator','Questioner (Debate 2)') # just for reference (see notes)
colors = c('black','purple')
```

The following code produces second-by-second event markers (0/1) for given events. For example, the speechturn variable specifies that someone is talking during that time; who is a vector that specifies a code (see keys below) marking who is doing that talking. The time codes for the interaction specify a time interval that is used (end - start) to determine how many seconds have to be inserted into the vectors.

```
timecodes$startt = round(timecodes$startt) # seconds can refer to rows, so let's round
timecodes$endt = round(timecodes$endt)
ixes = c() # which seconds to extract out of twitter data
speechturn = c() # which speech turn is it? used as random factor
who = c() # who is talking (see notes below)
utttime = c() # seconds WITHIN turn (e.g., 0 - 72, in 72s turn)

for (j in 2:nrow(timecodes)) { # omit first contribution, always moderator
    ixes = c(ixes,timecodes[j,]$startt:timecodes[j,]$endt) # get second range for this turn
    speechturn = c(speechturn,(timecodes[j,]$startt:timecodes[j,]$endt)*0+timecodes[j,]$speechturn) # w
    who = c(who,(timecodes[j,]$startt:timecodes[j,]$endt)*0+timecodes[j,]$who) # who is speaking?
```

```
    utttime = c(utttime,(timecodes[j,]$startt:timecodes[j,]$endt)-timecodes[j,]$startt); # order time w
}
who = as.factor(who) # make sure it's a factor
utttimeC = utttime - mean(utttime) # center for interaction term
```

The initial very simple analysis shows that tweets mention the speaker more as that speaker begins to talk. Results are quite robust across all debates.

```
dat = debate[ixes,]
utttimeC = utttimeC/sd(utttime) # standardize for betas
dv = (dat$obama/dat$total - mean(dat$obama/dat$total))/sd(dat$obama/dat$total)
lmo_obama = lmer(dv~(who==2)*utttimeC+(1+(who==2)+utttimeC|speechturn),data=debate[ixes,],REML=F) # max
coefs_obama = data.frame(summary(lmo_obama)$coefficients)
coefs_obama$p = 2*(1-pnorm(abs(coefs_obama$t.value)))
print(coefs_obama)
```

```
##                         Estimate Std..Error t.value          p
## (Intercept)              -0.3798    0.08473  -4.483 7.375e-06
## who == 2TRUE              0.7530    0.16455   4.576 4.733e-06
## utttimeC                 -0.2984    0.06977  -4.277 1.893e-05
## who == 2TRUE:utttimeC     0.6952    0.11027   6.305 2.880e-10
```

```
r.squaredGLMM(lmo_obama)
```

```
##    R2m    R2c
## 0.1974 0.7293
```

```
ccff = ccf((who==2),dv,200)
```

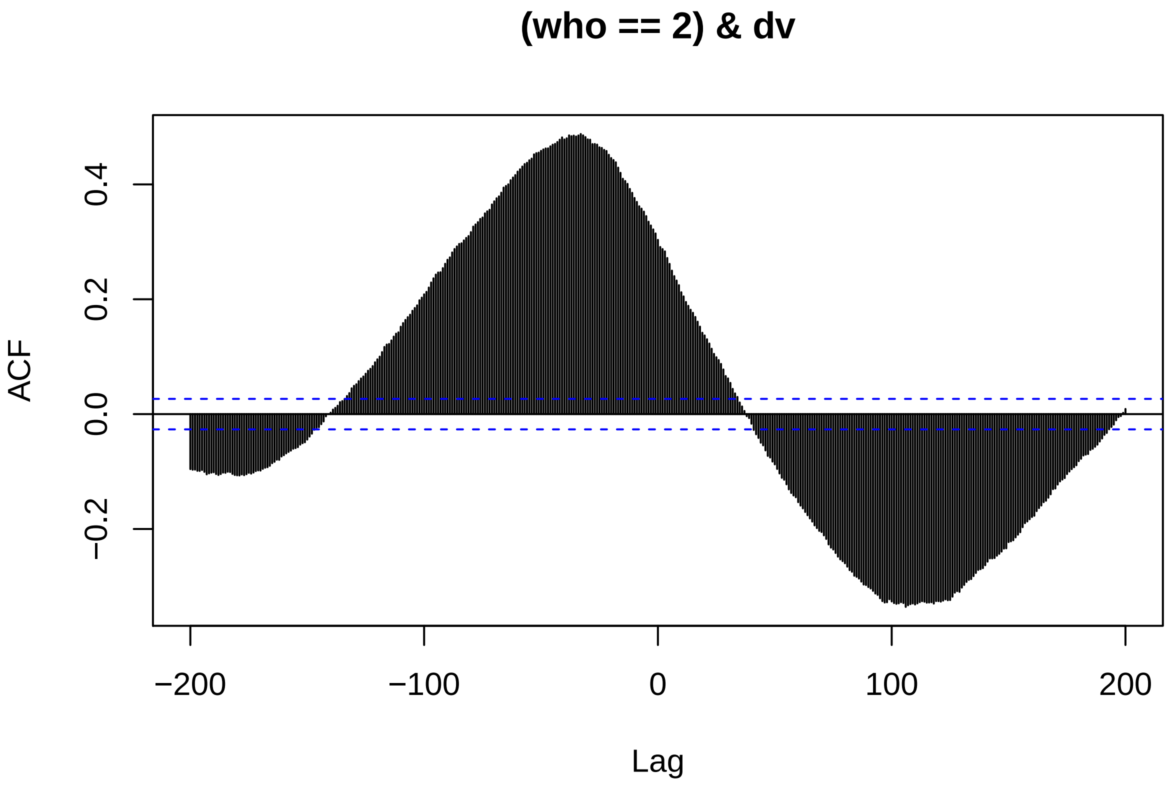

```r
dv = (dat$romney/dat$total - mean(dat$romney/dat$total))/sd(dat$romney/dat$total)
lmo_romney = lmer(dv~(who==1)*utttimeC+(1+(who==1)+utttimeC|speechturn),data=debate[ixes,],REML=F) # ma
coefs_romney = data.frame(summary(lmo_romney)$coefficients)
coefs_romney$p = 2*(1-pnorm(abs(coefs_romney$t.value)))
print(coefs_romney)
```

```
##                        Estimate Std..Error t.value         p
## (Intercept)             -0.2931    0.11847  -2.474 1.335e-02
## who == 1TRUE             0.6745    0.14004   4.817 1.459e-06
## utttimeC                -0.3657    0.07165  -5.104 3.327e-07
## who == 1TRUE:utttimeC    0.6900    0.09306   7.415 1.219e-13
```

```r
r.squaredGLMM(lmo_romney)
```

```
##    R2m    R2c
## 0.1549 0.7515
```

```r
ccff = ccf((who==1),dv,200)
```

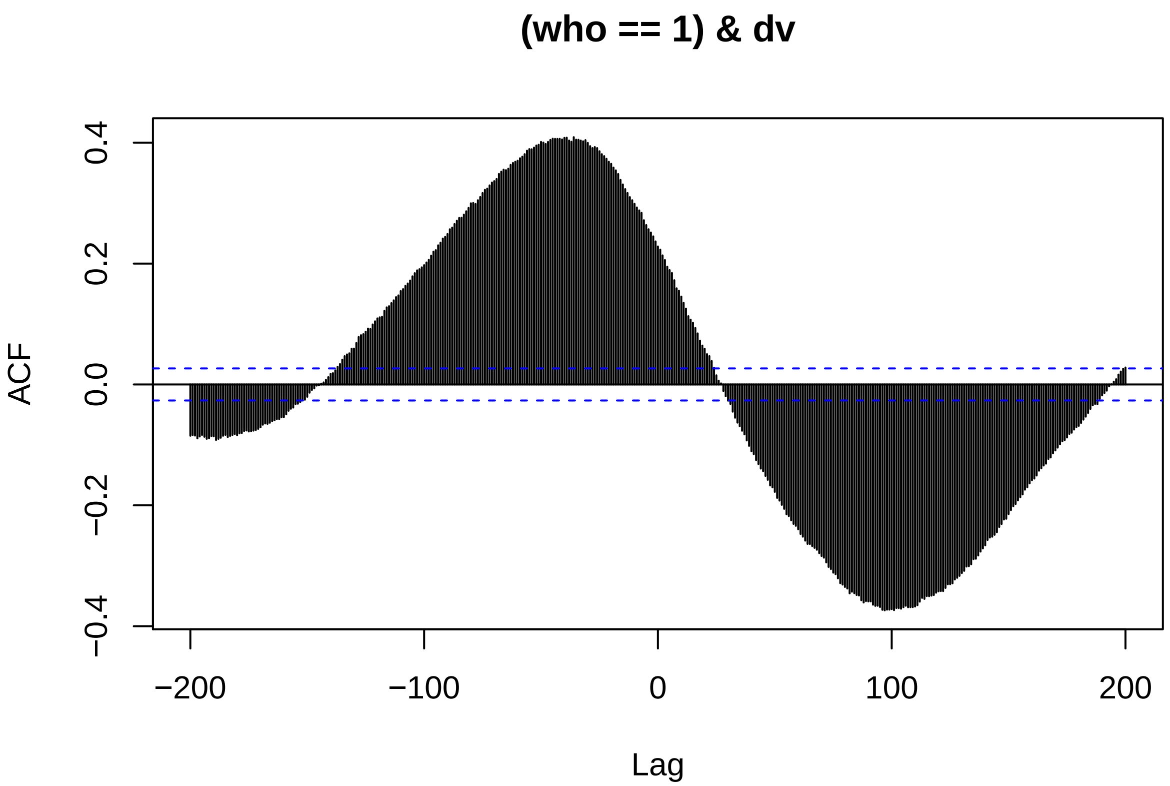


The following plots the Twitter intensity (mentions) along the y-axis and time into a turn, along the x-axis. All debates show evidence for this rapid shift of mentions, in just matters of seconds. Red is for Romney; blue for Obama. Grey lines reflect mentions to Romny/Obama *when anyone else is doing the talking.*

```r
library(ggplot2)
ggplot(debate[ixes,],aes(utttime,obama/total,color=(who==2)))+
  stat_summary(fun.data=mean_se,geom="pointrange",size=.5)+
    coord_cartesian(xlim=c(0,100),ylim=c(.2,.8))+
```

```
    scale_color_manual(values=c('gray','blue'))+
    scale_y_continuous(breaks=c(.25,.5,.75))+
    scale_x_continuous(breaks=c(0,50,100))+
    xlab("")+ylab("")
```

```
## Warning: Removed 56 rows containing missing values (geom_segment).
## Warning: Removed 12 rows containing missing values (geom_segment).
```

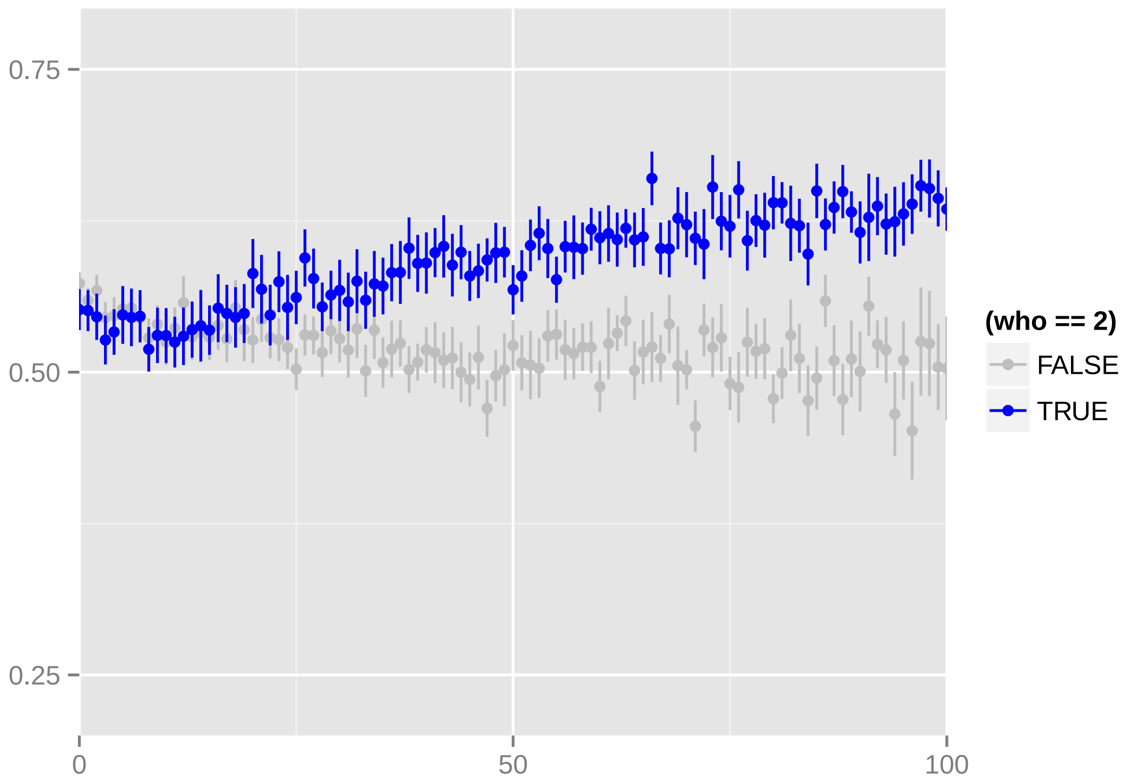


```
ggplot(debate[ixes,],aes(utttime,romney/total,color=(who==1)))+
    stat_summary(fun.data=mean_se,geom="pointrange",size=.5)+
    coord_cartesian(xlim=c(0,100),ylim=c(.2,.8))+
    scale_color_manual(values=c('gray','red'))+
    scale_y_continuous(breaks=c(.25,.5,.75))+
    scale_x_continuous(breaks=c(0,50,100))+
    xlab("")+ylab("")
```

```
## Warning: Removed 12 rows containing missing values (geom_segment).
## Warning: Removed 56 rows containing missing values (geom_segment).
```

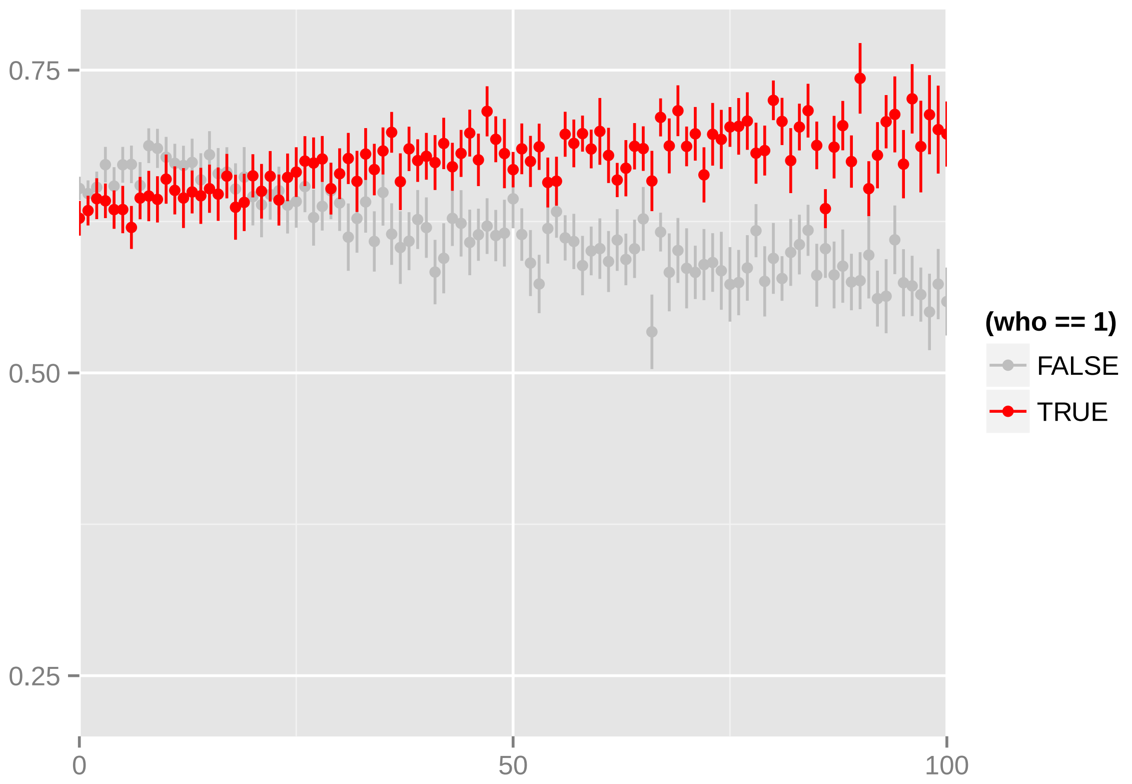


The following is an important set of information regarding the data coding and analysis.

Debate 1 starts in transcripts: 9:01:44.

Debate 2 starts in transcripts: 9:01:49.

Debate 3 starts in transcripts: 9:01:52

Timed using stopwatch + CSPAN clock – at the onset of speech.

From looking into the video / transcript:

big bird: 26:11

binders full of women 38:44

[horses and ]bayonets 42:03

For the time codes:

3-Interlocutor (1=Romney, 2=Obama, 3=Moderator, 4=Questioner in debate2)

6-Interruptions1 (1=interruption, 2=interrupting and taking the ground)

7-Interruptions2 (1=Moderator interrupts Romney, 2=M interrupts O, 3=O interrupts R, 4=R interrupts O, 5=R interrupts M, 6=O interrupts M)

# A breakdown of all data and the data fusion process, along with initial speaker analysis (Debate 2)

First, we load in required libraries and specify variables that were determined in order to time-align the Twitter and CSPAN feeds. CSPAN times were determined by the by-the-second debate initiation using real time. Token mention is the time, in seconds, at which the salient moment was introduced in the debate (see the paper for more details).

```
library(MuMIn)
library(lme4)
library(MuMIn)
library(languageR)
library(ggplot2)

token_transcript_mention = c(26*60+11,38*60+44,42*60+3) # time when tokens (salient events) mentioned
transcripts_time_starts = c('Wed Oct  3 21:01:44 2012','Tue Oct 16 21:01:49 2012','Mon Oct 22 21:01:52 
```

Next, we determine which debate we are analyzing, load in the relevant files, and time-align the Twitter and conversation dynamics. "nort" means that the Twitter data reflects tweets without any retweets.

```
dnum = 2 # debate number

debate = read.table(paste('../data/debate',dnum,'.nort.txt',sep=''),head=TRUE,sep='\t') # load in debat
colnames(debate) = c('date','total','obama','romney','token')

### let's align the debate twitter data
align_index = which(debate$date==transcripts_time_starts[dnum]) # get row number where debate time star
debate = debate[align_index:nrow(debate),] # start debate data from second of debate onset

timecodes = read.table('../data/timecodes.txt',sep='\t',head=FALSE) # time codes from aarhus
colnames(timecodes) = c('startt','endt','who','speechturn','dnum','interrupt1','interrupt2','topic')
timecodes = timecodes[timecodes$dnum==dnum,] # take only the current debate

speakers = c('Romney','Obama','Moderator','Questioner (Debate 2)') # just for reference (see notes)
colors = c('black','purple')
```

The following code produces second-by-second event markers (0/1) for given events. For example, the speechturn variable specifies that someone is talking during that time; who is a vector that specifies a code (see keys below) marking who is doing that talking. The time codes for the interaction specify a time interval that is used (end - start) to determine how many seconds have to be inserted into the vectors.

```
timecodes$startt = round(timecodes$startt) # seconds can refer to rows, so let's round
timecodes$endt = round(timecodes$endt)
ixes = c() # which seconds to extract out of twitter data
speechturn = c() # which speech turn is it? used as random factor
who = c() # who is talking (see notes below)
utttime = c() # seconds WITHIN turn (e.g., 0 - 72, in 72s turn)

for (j in 2:nrow(timecodes)) { # omit first contribution, always moderator
    ixes = c(ixes,timecodes[j,]$startt:timecodes[j,]$endt) # get second range for this turn
    speechturn = c(speechturn,(timecodes[j,]$startt:timecodes[j,]$endt)*0+timecodes[j,]$speechturn) # w
```

```
    who = c(who,(timecodes[j,]$startt:timecodes[j,]$endt)*0+timecodes[j,]$who) # who is speaking?
    utttime = c(utttime,(timecodes[j,]$startt:timecodes[j,]$endt)-timecodes[j,]$startt); # order time w
}
who = as.factor(who) # make sure it's a factor
utttimeC = utttime - mean(utttime) # center for interaction term
```

The initial very simple analysis shows that tweets mention the speaker more as that speaker begins to talk. Results are quite robust across all debates.

```
dat = data=debate[ixes,]
utttimeC = utttimeC/sd(utttime) # standardize for betas
dv = (dat$obama/dat$total - mean(dat$obama/dat$total))/sd(dat$obama/dat$total)
lmo_obama = lmer(dv~(who==2)*utttimeC+(1+(who==2)+utttimeC|speechturn),data=debate[ixes,],REML=F) # max
coefs_obama = data.frame(summary(lmo_obama)$coefficients)
coefs_obama$p = 2*(1-pnorm(abs(coefs_obama$t.value)))
print(coefs_obama)
```

```
##                         Estimate Std..Error t.value         p
## (Intercept)              -0.4388    0.08941  -4.907 9.226e-07
## who == 2TRUE              1.1562    0.13408   8.623 0.000e+00
## utttimeC                 -0.4964    0.07323  -6.778 1.217e-11
## who == 2TRUE:utttimeC     0.9837    0.10820   9.092 0.000e+00
```

```
r.squaredGLMM(lmo_obama)
```

```
##    R2m    R2c
## 0.3878 0.8209
```

```
dv = (dat$romney/dat$total - mean(dat$romney/dat$total))/sd(dat$romney/dat$total)
lmo_romney = lmer(dv~(who==1)*utttimeC+(1+(who==1)+utttimeC|speechturn),data=debate[ixes,],REML=F) # ma
coefs_romney = data.frame(summary(lmo_romney)$coefficients)
coefs_romney$p = 2*(1-pnorm(abs(coefs_romney$t.value)))
print(coefs_romney)
```

```
##                         Estimate Std..Error t.value         p
## (Intercept)              -0.5537    0.09464  -5.851 4.896e-09
## who == 1TRUE              1.0389    0.12676   8.196 2.220e-16
## utttimeC                 -0.4192    0.07814  -5.366 8.070e-08
## who == 1TRUE:utttimeC     0.9793    0.10765   9.097 0.000e+00
```

```
r.squaredGLMM(lmo_romney)
```

```
##    R2m    R2c
## 0.3579 0.7795
```

The following plots the Twitter intensity (mentions) along the y-axis and time into a turn, along the x-axis. All debates show evidence for this rapid shift of mentions, in just matters of seconds. Red is for Romney; blue for Obama. Grey lines reflect mentions to Romny/Obama *when anyone else is doing the talking.*

```
library(ggplot2)
ggplot(debate[ixes,],aes(utttime,obama/total,color=(who==2)))+
  stat_summary(fun.data=mean_se,geom="pointrange",size=.5)+
    coord_cartesian(xlim=c(0,100),ylim=c(.2,.8))+
    scale_color_manual(values=c('gray','blue'))+
    scale_y_continuous(breaks=c(.25,.5,.75))+
    scale_x_continuous(breaks=c(0,50,100))+
    xlab("")+ylab("")
```

```
## Warning: Removed 10 rows containing missing values (geom_segment).
## Warning: Removed 34 rows containing missing values (geom_segment).
```

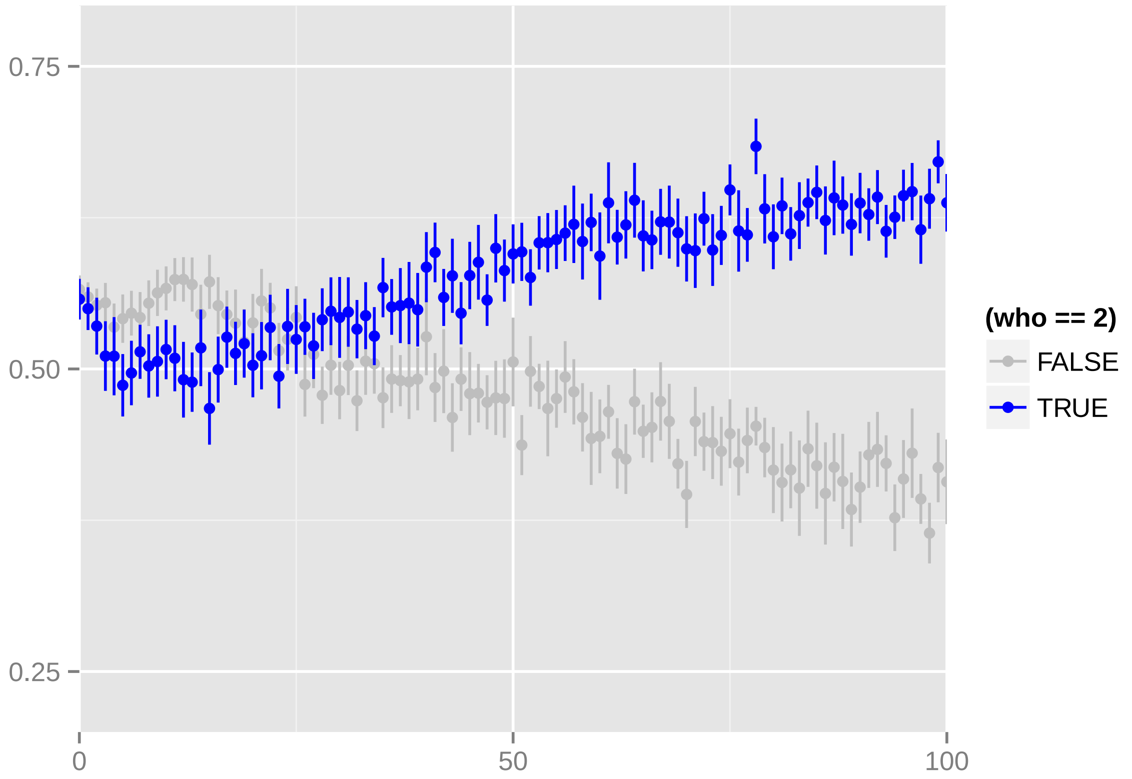


```
ggplot(debate[ixes,],aes(utttime,romney/total,color=(who==1)))+
    stat_summary(fun.data=mean_se,geom="pointrange",size=.5)+
    coord_cartesian(xlim=c(0,100),ylim=c(.2,.8))+
    scale_color_manual(values=c('gray','red'))+
    scale_y_continuous(breaks=c(.25,.5,.75))+
    scale_x_continuous(breaks=c(0,50,100))+
    xlab("")+ylab("")
```

```
## Warning: Removed 34 rows containing missing values (geom_segment).
## Warning: Removed 10 rows containing missing values (geom_segment).
```

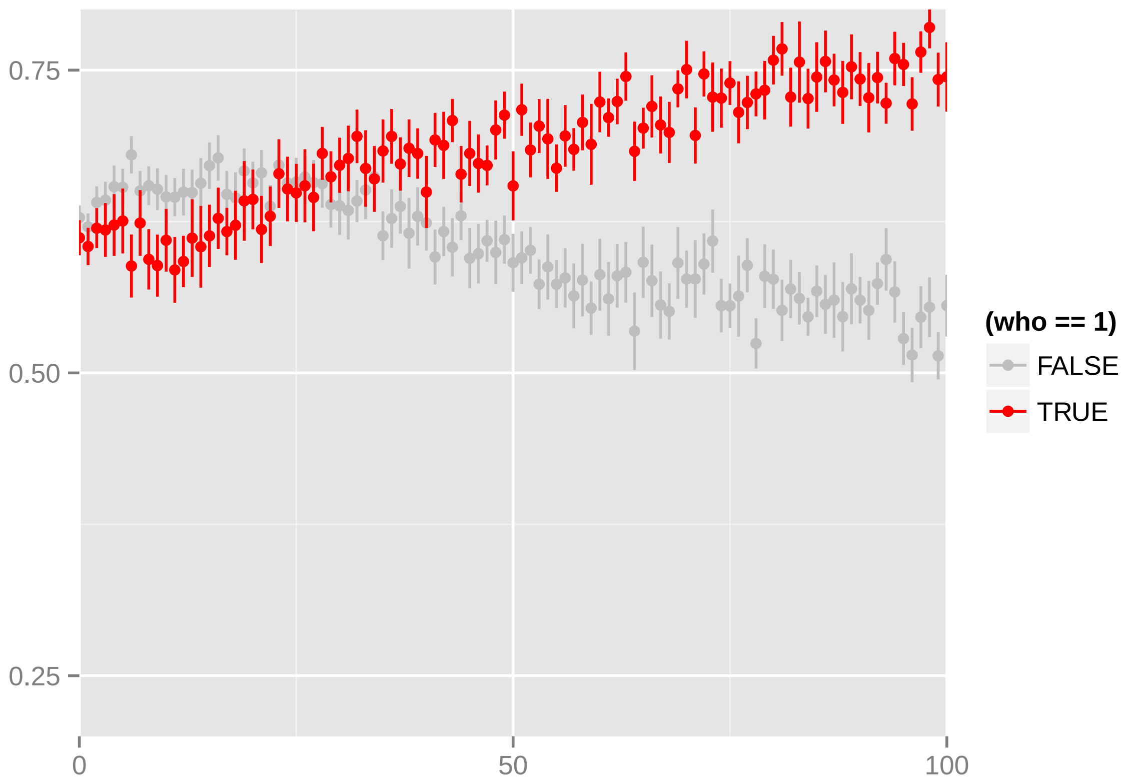


The following is an important set of information regarding the data coding and analysis.

Debate 1 starts in transcripts: 9:01:44.

Debate 2 starts in transcripts: 9:01:49.

Debate 3 starts in transcripts: 9:01:52

Timed using stopwatch + CSPAN clock – at the onset of speech.

From looking into the video / transcript:

big bird: 26:11

binders full of women 38:44

[horses and ]bayonets 42:03

For the time codes:

3-Interlocutor (1=Romney, 2=Obama, 3=Moderator, 4=Questioner in debate2)

6-Interruptions1 (1=interruption, 2=interrupting and taking the ground)

7-Interruptions2 (1=Moderator interrupts Romney, 2=M interrupts O, 3=O interrupts R, 4=R interrupts O, 5=R interrupts M, 6=O interrupts M)

# A breakdown of all data and the data fusion process, along with initial speaker analysis (Debate 3)

First, we load in required libraries and specify variables that were determined in order to time-align the Twitter and CSPAN feeds. CSPAN times were determined by the by-the-second debate initiation using real time. Token mention is the time, in seconds, at which the salient moment was introduced in the debate (see the paper for more details).

```
library(MuMIn)
library(lme4)
library(languageR)
library(ggplot2)

token_transcript_mention = c(26*60+11,38*60+44,42*60+3) # time when tokens (salient events) mentioned
transcripts_time_starts = c('Wed Oct  3 21:01:44 2012','Tue Oct 16 21:01:49 2012','Mon Oct 22 21:01:52 2
```

Next, we determine which debate we are analyzing, load in the relevant files, and time-align the Twitter and conversation dynamics. "nort" means that the Twitter data reflects tweets without any retweets.

```
dnum = 3 # debate number

debate = read.table(paste('../data/debate',dnum,'.nort.txt',sep=''),head=TRUE,sep='\t') # load in debat
colnames(debate) = c('date','total','obama','romney','token')

### let's align the debate twitter data
align_index = which(debate$date==transcripts_time_starts[dnum]) # get row number where debate time star
debate = debate[align_index:nrow(debate),] # start debate data from second of debate onset

timecodes = read.table('../data/timecodes.txt',sep='\t',head=FALSE) # time codes from aarhus
colnames(timecodes) = c('startt','endt','who','speechturn','dnum','interrupt1','interrupt2','topic')
timecodes = timecodes[timecodes$dnum==dnum,] # take only the current debate

speakers = c('Romney','Obama','Moderator','Questioner (Debate 2)') # just for reference (see notes)
colors = c('black','purple')
```

The following code produces second-by-second event markers (0/1) for given events. For example, the speechturn variable specifies that someone is talking during that time; who is a vector that specifies a code (see keys below) marking who is doing that talking. The time codes for the interaction specify a time interval that is used (end - start) to determine how many seconds have to be inserted into the vectors.

```
timecodes$startt = round(timecodes$startt) # seconds can refer to rows, so let's round
timecodes$endt = round(timecodes$endt)
ixes = c() # which seconds to extract out of twitter data
speechturn = c() # which speech turn is it? used as random factor
who = c() # who is talking (see notes below)
utttime = c() # seconds WITHIN turn (e.g., 0 - 72, in 72s turn)

for (j in 2:nrow(timecodes)) { # omit first contribution, always moderator
    ixes = c(ixes,timecodes[j,]$startt:timecodes[j,]$endt) # get second range for this turn
    speechturn = c(speechturn,(timecodes[j,]$startt:timecodes[j,]$endt)*0+timecodes[j,]$speechturn) # w
    who = c(who,(timecodes[j,]$startt:timecodes[j,]$endt)*0+timecodes[j,]$who) # who is speaking?
```

```
    utttime = c(utttime,(timecodes[j,]$startt:timecodes[j,]$endt)-timecodes[j,]$startt); # order time w
}
who = as.factor(who) # make sure it's a factor
utttimeC = utttime - mean(utttime) # center for interaction term
```

The initial very simple analysis shows that tweets mention the speaker more as that speaker begins to talk. Results are quite robust across all debates.

```
dat = data=debate[ixes,]
utttimeC = utttimeC/sd(utttime) # standardize for betas
dv = (dat$obama/dat$total - mean(dat$obama/dat$total))/sd(dat$obama/dat$total)
lmo_obama = lmer(dv~(who==2)*utttimeC+(1+(who==2)+utttimeC|speechturn),data=debate[ixes,],REML=F) # max
```

```
## Warning: Model failed to converge: degenerate Hessian with 1 negative
## eigenvalues
```

```
coefs_obama = data.frame(summary(lmo_obama)$coefficients)
coefs_obama$p = 2*(1-pnorm(abs(coefs_obama$t.value)))
print(coefs_obama)
```

```
##                        Estimate Std..Error t.value         p
## (Intercept)             -0.2057    0.09586  -2.146 3.185e-02
## who == 2TRUE             0.4667    0.13681   3.412 6.460e-04
## utttimeC                -0.2082    0.07677  -2.712 6.687e-03
## who == 2TRUE:utttimeC    0.4961    0.11438   4.337 1.443e-05
```

```
r.squaredGLMM(lmo_obama)
```

```
##    R2m    R2c
## 0.1076 0.5845
```

```
ccff = ccf((who==2),dv,200)
```

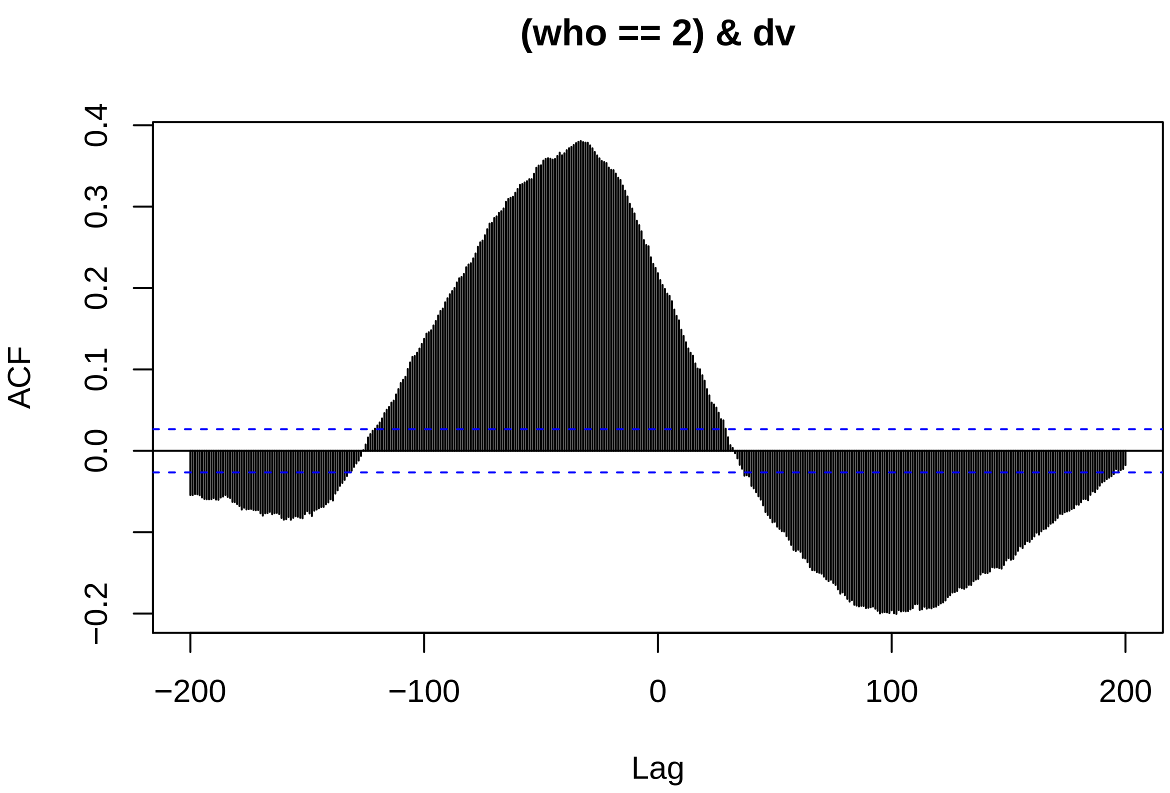


```
dv = (dat$romney/dat$total - mean(dat$romney/dat$total))/sd(dat$romney/dat$total)
lmo_romney = lmer(dv~(who==1)*utttimeC+(1+(who==1)+utttimeC|speechturn),data=debate[ixes,],REML=F) # ma
coefs_romney = data.frame(summary(lmo_romney)$coefficients)
coefs_romney$p = 2*(1-pnorm(abs(coefs_romney$t.value)))
print(coefs_romney)
```

```
##                       Estimate Std..Error t.value         p
## (Intercept)            -0.2583    0.09084  -2.843 4.470e-03
## who == 1TRUE            0.6037    0.13396   4.507 6.582e-06
## utttimeC               -0.2290    0.07336  -3.121 1.802e-03
## who == 1TRUE:utttimeC   0.4268    0.10763   3.965 7.330e-05
```

```
r.squaredGLMM(lmo_romney)
```

```
##    R2m    R2c
## 0.1232 0.5665
```

```
ccff = ccf((who==1),dv,200)
```

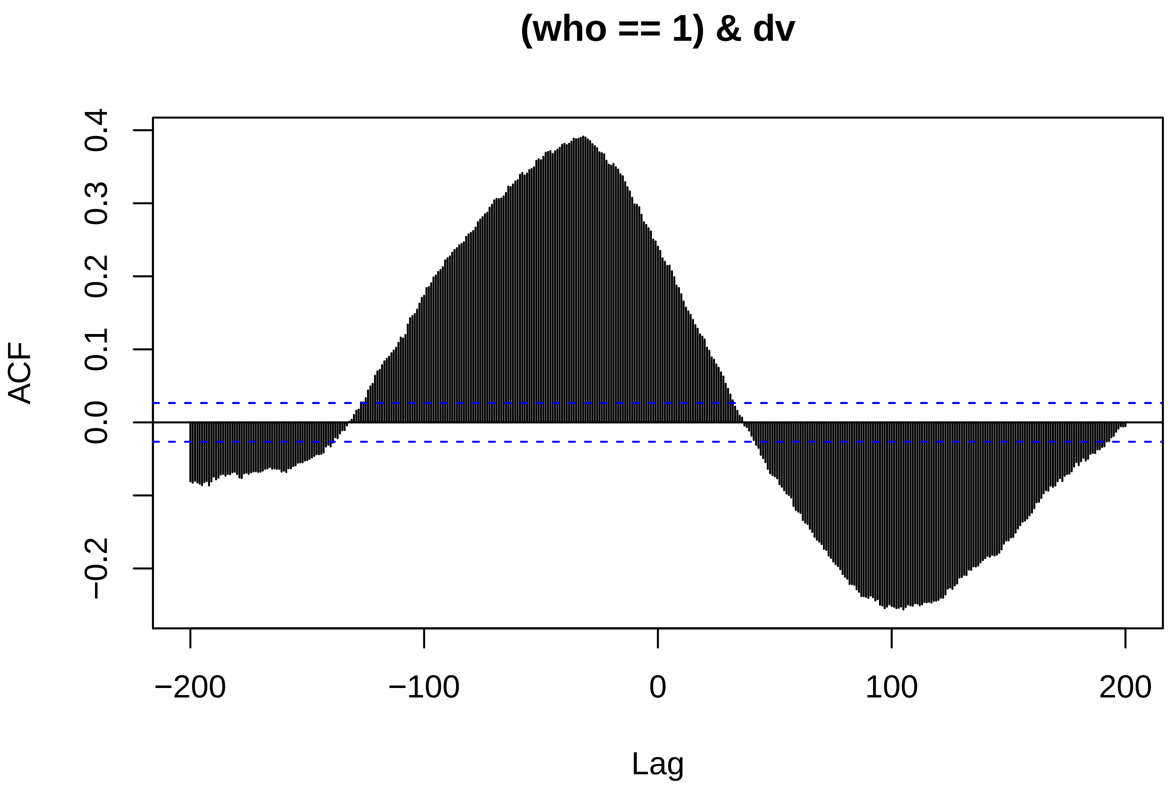


The following plots the Twitter intensity (mentions) along the y-axis and time into a turn, along the x-axis. All debates show evidence for this rapid shift of mentions, in just matters of seconds. Red is for Romney; blue for Obama. Grey lines reflect mentions to Romny/Obama *when anyone else is doing the talking.*

```
library(ggplot2)
ggplot(debate[ixes,],aes(utttime,obama/total,color=(who==2)))+
    stat_summary(fun.data=mean_se,geom="pointrange",size=.5)+
    coord_cartesian(xlim=c(0,100),ylim=c(.2,.8))+
    scale_color_manual(values=c('gray','blue'))+
    scale_y_continuous(breaks=c(.25,.5,.75))+
    scale_x_continuous(breaks=c(0,50,100))+
    xlab("")+ylab("")
```

```
## Warning: Removed 1 rows containing missing values (geom_segment).
## Warning: Removed 5 rows containing missing values (geom_segment).
```

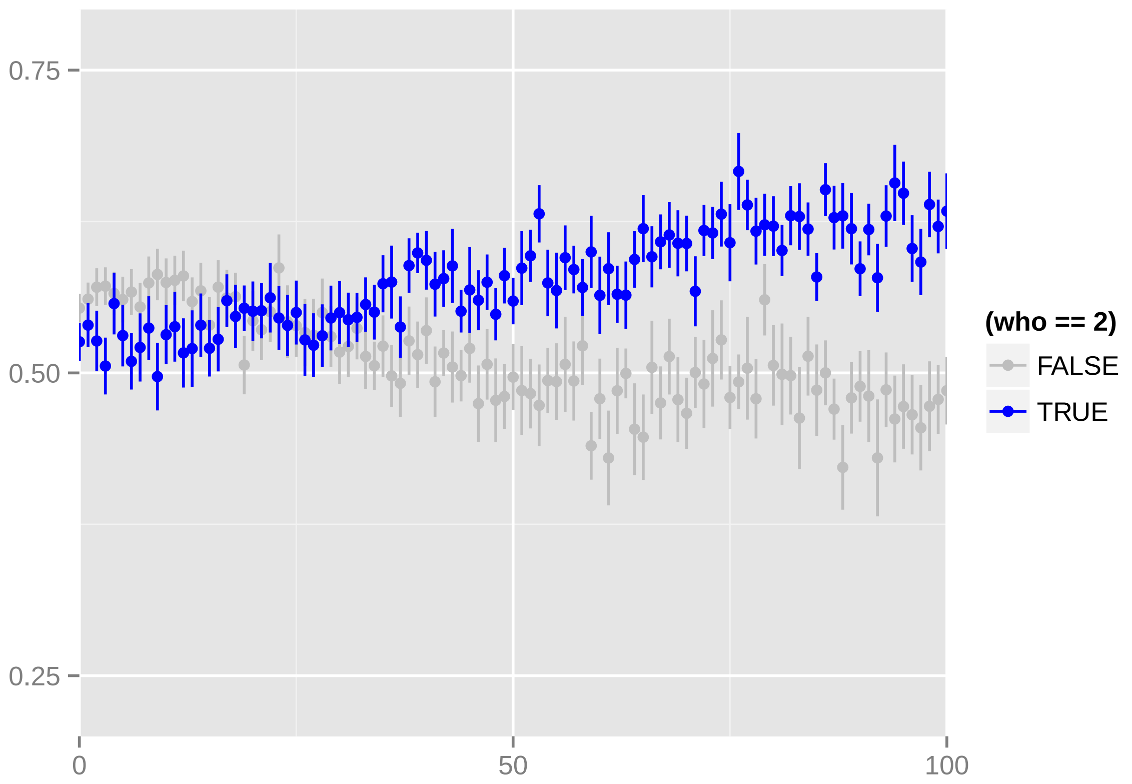


```
ggplot(debate[ixes,],aes(utttime,romney/total,color=(who==1)))+
    stat_summary(fun.data=mean_se,geom="pointrange",size=.5)+
    coord_cartesian(xlim=c(0,100),ylim=c(.2,.8))+
    scale_color_manual(values=c('gray','red'))+
    scale_y_continuous(breaks=c(.25,.5,.75))+
    scale_x_continuous(breaks=c(0,50,100))+
    xlab("")+ylab("")
```

```
## Warning: Removed 5 rows containing missing values (geom_segment).
## Warning: Removed 1 rows containing missing values (geom_segment).
```

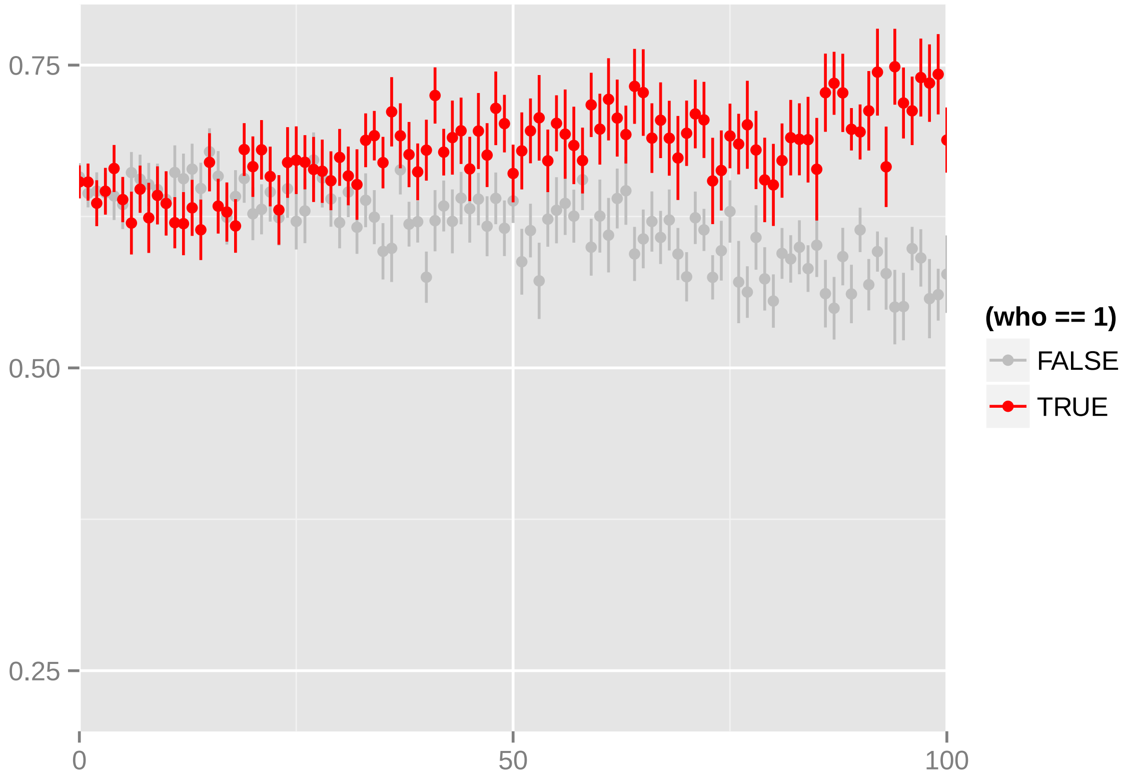


The following is an important set of information regarding the data coding and analysis.

Debate 1 starts in transcripts: 9:01:44.

Debate 2 starts in transcripts: 9:01:49.

Debate 3 starts in transcripts: 9:01:52

Timed using stopwatch + CSPAN clock – at the onset of speech.

From looking into the video / transcript:

big bird: 26:11

binders full of women 38:44

[horses and ]bayonets 42:03

For the time codes:

3-Interlocutor (1=Romney, 2=Obama, 3=Moderator, 4=Questioner in debate2)

6-Interruptions1 (1=interruption, 2=interrupting and taking the ground)

7-Interruptions2 (1=Moderator interrupts Romney, 2=M interrupts O, 3=O interrupts R, 4=R interrupts O, 5=R interrupts M, 6=O interrupts M)

# Influence on tweet rate by interruptions (Debate 1)

We load libraries and prepare parameters, as other *.Rmd's specify. This block also includes the alignment of data.

```
library(MuMIn)
library(lme4)
library(languageR)
library(ggplot2)

token_transcript_mention = c(26*60+11,38*60+44,42*60+3) # time when tokens (salient events) mentioned
transcripts_time_starts = c('Wed Oct  3 21:01:44 2012','Tue Oct 16 21:01:49 2012','Mon Oct 22 21:01:52 

dnum = 1 # debate number

debate = read.table(paste('../data/debate',dnum,'.nort.txt',sep=''),head=TRUE,sep='\t') # load in debat
colnames(debate) = c('date','total','obama','romney','token')

### let's align the debate twitter data
align_index = which(debate$date==transcripts_time_starts[dnum]) # get row number where debate time star
debate = debate[align_index:nrow(debate),] # start debate data from second of debate onset

timecodes = read.table('../data/timecodes.txt',sep='\t',head=FALSE) # time codes from aarhus
colnames(timecodes) = c('startt','endt','who','speechturn','dnum','interrupt1','interrupt2','topic')
timecodes = timecodes[timecodes$dnum==dnum,] # take only the current debate

speakers = c('Romney','Obama','Moderator','Questioner (Debate 2)') # just for reference (see notes)
colors = c('black','purple')
```

In the next block, we determine the intervals which involve any form of interruption. Get some descriptives, too.

```
dim(timecodes)[1]
```

```
## [1] 214
```

```
sum(timecodes$interrupt1>0)
```

```
## [1] 115
```

```
ints = timecodes[timecodes$interrupt1>0,]
min(ints$endt-ints$startt) # shortest interruption
```

```
## [1] 0.1
```

```
max(ints$endt-ints$startt) # longest interruption
```

```
## [1] 130.6
```

```r
table(ints$who) # who interrupts whom here?
```

```
##
##  1  2  3
## 45 23 47
```

Next we use these intervals to create predictors based on the sequence of interruptions (second-by-second).

```r
# indices included in the analysis (reflecting # of seconds into
# debate, with debate array starting at 0, so can index using row #)
ixes = c()  # seconds / rows to be used to inspect twitter data (seconds can reference rows, since deba
inutt = c() # the time that the turn has been going on
interrupt = c() # whether a turn is an interruption
speechturn = c() # unique segment #, as random factor, to ensure not driven by small # of utterances

timecodes$endt = round(timecodes$endt) # can be used to reference debate directly, need integers
timecodes$startt = round(timecodes$startt)

for (j in 2:nrow(timecodes)) { # moderator always 1, skip
    ixes = c(ixes,(timecodes[j,]$startt:timecodes[j,]$endt)) # get range of s
    inutt = c(inutt,(timecodes[j,]$startt:timecodes[j,]$endt)-timecodes[j,]$startt)
    speechturn = c(speechturn,(timecodes[j,]$startt:timecodes[j,]$endt)*0+timecodes[j,]$speechturn)
    # is this an interruption
    interrupt = c(interrupt,(timecodes[j,]$startt:timecodes[j,]$endt)*0+(timecodes[j,]$interrupt1>0)*1)
}
```

Now that we have our predictors we can run full lmer models to determine whether interruptions contribute to tweet rate. We then plot.

```r
# check then use fully saturated (maximal) random effect structure within speech turns
intC = interrupt # keep it dichotomous; results consistent, just change utt coefficient (uninteresting)
inuttC = (inutt - mean(inutt))/sd(inutt)
dv = (debate[ixes,]$total - mean(debate[ixes,]$total))/sd(debate[ixes,]$total)
lmo = lmer(dv~intC*inuttC+(1+intC*inuttC|speechturn),data=debate[ixes,])
coefs = data.frame(summary(lmo)$coefficients)
coefs$p = 2*(1-pnorm(abs(coefs$t.value)))
print(coefs)
```

```
##              Estimate Std..Error t.value         p
## (Intercept)  0.08253    0.09638  0.8563 0.3918533
## intC         0.71956    0.35982  1.9998 0.0455236
## inuttC      -0.20569    0.05417 -3.7974 0.0001462
## intC:inuttC  0.79712    0.32143  2.4799 0.0131409
```

```r
r.squaredGLMM(lmo)
```

```
##      R2m     R2c
## 0.07844 0.85115
```

```
# check for first 30 seconds (consistent)
lmo = lmer(debate[ixes[inutt<=30],]$total~intC[inutt<=30]*inuttC[inutt<=30]+(1+intC[inutt<=30]*inuttC[in
coefs = data.frame(summary(lmo)$coefficients)
coefs$p = 2*(1-pnorm(abs(coefs$t.value)))
print(coefs)
```

```
##                                       Estimate Std..Error t.value       p
## (Intercept)                             58.710      2.761  21.265 0.00000
## intC[inutt <= 30]                       13.716      6.578   2.085 0.03705
## inuttC[inutt <= 30]                     -4.255      2.335  -1.822 0.06842
## intC[inutt <= 30]:inuttC[inutt <= 30]   14.965      5.955   2.513 0.01197
```

```
library(ggplot2)
# debate 1
ggplot(debate[ixes,],aes(inutt,total,color=interrupt>0))+
    stat_summary(fun.data=mean_se,geom="pointrange",size=.5)+
    coord_cartesian(xlim=c(0,30),ylim=c(50,75))+
    scale_color_manual(values=colors)+
    scale_x_continuous(breaks=c(0,10,20,30,40,50,60))+
    scale_y_continuous(breaks=c(50,55,60,65,70,75))+
    xlab("")+ylab("")
```

```
## Warning: Removed 10 rows containing missing values (geom_segment).
## Warning: Removed 12 rows containing missing values (geom_segment).
```

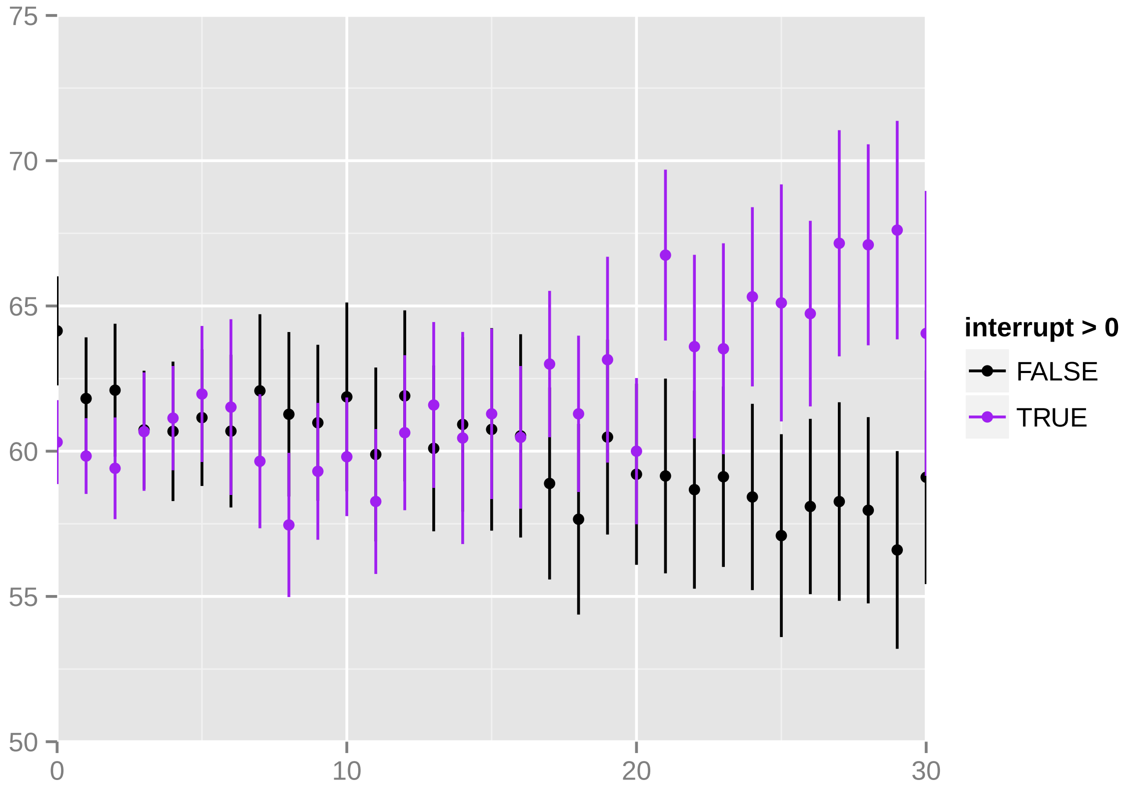

```r
ccff = ccf(interrupt,dv,200)
```

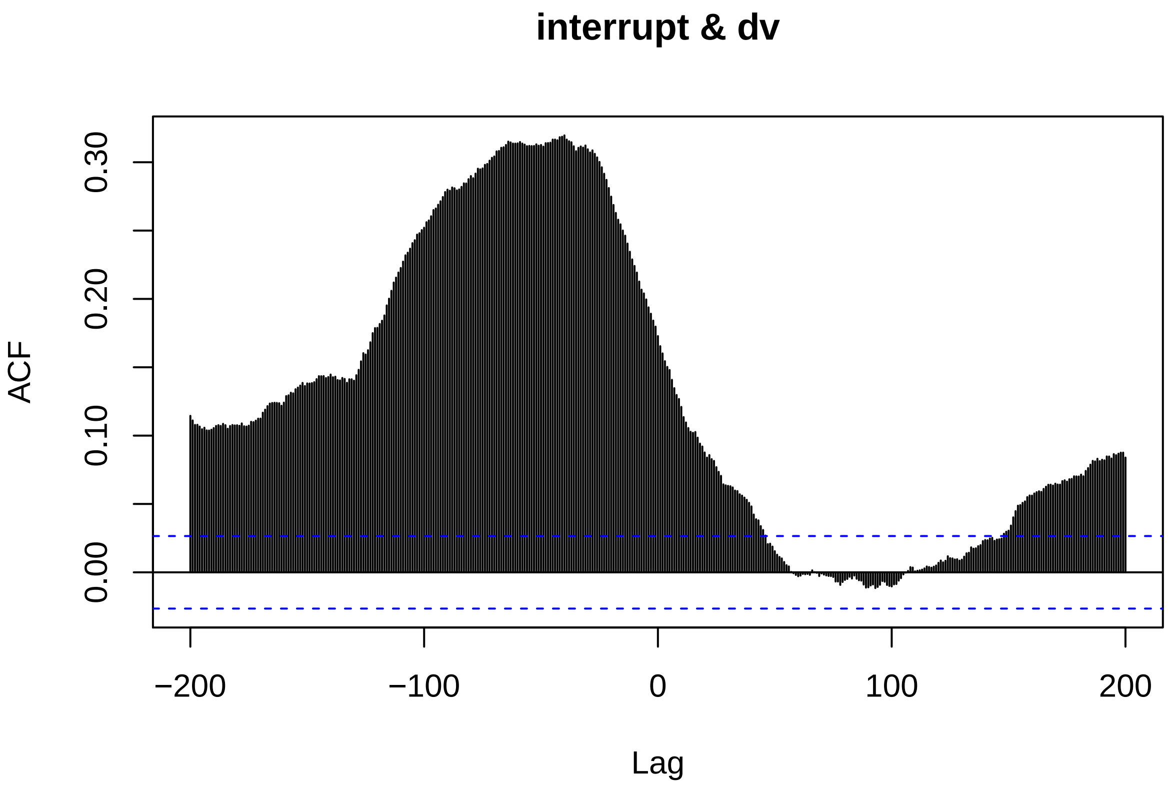


The following is an important set of information regarding the data coding and analysis.

Debate 1 starts in transcripts: 9:01:44.

Debate 2 starts in transcripts: 9:01:49.

Debate 3 starts in transcripts: 9:01:52

Timed using stopwatch + CSPAN clock – at the onset of speech.

From looking into the video / transcript:

big bird: 26:11

binders full of women 38:44

[horses and ]bayonets 42:03

For the time codes:

3-Interlocutor (1=Romney, 2=Obama, 3=Moderator, 4=Questioner in debate2)

6-Interruptions1 (1=interruption, 2=interrupting and taking the ground)

7-Interruptions2 (1=Moderator interrupts Romney, 2=M interrupts O, 3=O interrupts R, 4=R interrupts O, 5=R interrupts M, 6=O interrupts M)

# Influence on tweet rate by interruptions (Debate 2)

We load libraries and prepare parameters, as other *.Rmd's specify. This block also includes the alignment of data.

```
library(MuMIn)
library(lme4)
library(languageR)
library(ggplot2)

token_transcript_mention = c(26*60+11,38*60+44,42*60+3) # time when tokens (salient events) mentioned
transcripts_time_starts = c('Wed Oct  3 21:01:44 2012','Tue Oct 16 21:01:49 2012','Mon Oct 22 21:01:52 

dnum = 2 # debate number

debate = read.table(paste('../data/debate',dnum,'.nort.txt',sep=''),head=TRUE,sep='\t') # load in debat
colnames(debate) = c('date','total','obama','romney','token')

### let's align the debate twitter data
align_index = which(debate$date==transcripts_time_starts[dnum]) # get row number where debate time star
debate = debate[align_index:nrow(debate),] # start debate data from second of debate onset

timecodes = read.table('../data/timecodes.txt',sep='\t',head=FALSE) # time codes from aarhus
colnames(timecodes) = c('startt','endt','who','speechturn','dnum','interrupt1','interrupt2','topic')
timecodes = timecodes[timecodes$dnum==dnum,] # take only the current debate

speakers = c('Romney','Obama','Moderator','Questioner (Debate 2)') # just for reference (see notes)
colors = c('black','purple')
```

In the next block, we determine the intervals which involve any form of interruption. Get some descriptives, too.

```
dim(timecodes)[1]
```

```
## [1] 266
```

```
sum(timecodes$interrupt1>0)
```

```
## [1] 105
```

```
ints = timecodes[timecodes$interrupt1>0,]
min(ints$endt-ints$startt) # shortest interruption
```

```
## [1] 0
```

```
max(ints$endt-ints$startt) # longest interruption
```

```
## [1] 208.7
```

```
table(ints$who) # who interrupts whom here?
```

```
##
##  1  2  3
## 37 39 29
```

Next we use these intervals to create predictors based on the sequence of interruptions (second-by-second).

```
# indices included in the analysis (reflecting # of seconds into
# debate, with debate array starting at 0, so can index using row #)
ixes = c()  # seconds / rows to be used to inspect twitter data (seconds can reference rows, since deba
inutt = c() # the time that the turn has been going on
interrupt = c() # whether a turn is an interruption
speechturn = c() # unique segment #, as random factor, to ensure not driven by small # of utterances

timecodes$endt = round(timecodes$endt) # can be used to reference debate directly, need integers
timecodes$startt = round(timecodes$startt)

for (j in 2:nrow(timecodes)) { # moderator always 1, skip
    ixes = c(ixes,(timecodes[j,]$startt:timecodes[j,]$endt)) # get range of s
    inutt = c(inutt,(timecodes[j,]$startt:timecodes[j,]$endt)-timecodes[j,]$startt)
    speechturn = c(speechturn,(timecodes[j,]$startt:timecodes[j,]$endt)*0+timecodes[j,]$speechturn)
    # is this an interruption
    interrupt = c(interrupt,(timecodes[j,]$startt:timecodes[j,]$endt)*0+(timecodes[j,]$interrupt1>0)*1)
}
```

Now that we have our predictors we can run full lmer models to determine whether interruptions contribute to tweet rate. We then plot.

```
# check then use fully saturated (maximal) random effect structure within speech turns
intC = interrupt # keep it dichotomous; results consistent, just change utt coefficient (uninteresting)
inuttC = (inutt - mean(inutt))/sd(inutt)
dv = (debate[ixes,]$total - mean(debate[ixes,]$total))/sd(debate[ixes,]$total)
lmo = lmer(dv~intC*inuttC+(1+intC*inuttC|speechturn),data=debate[ixes,])
coefs = data.frame(summary(lmo)$coefficients)
coefs$p = 2*(1-pnorm(abs(coefs$t.value)))
print(coefs)
```

```
##              Estimate Std..Error t.value       p
## (Intercept)  0.31657     0.1377  2.2987 0.02152
## intC         0.55681     0.2237  2.4888 0.01282
## inuttC      -0.06901     0.1077 -0.6406 0.52181
## intC:inuttC  0.11414     0.1585  0.7200 0.47154
```

```
r.squaredGLMM(lmo)
```

```
##     R2m     R2c
## 0.01853 0.92696
```

```r
# check for first 30 seconds (consistent)
lmo = lmer(debate[ixes[inutt<=30],]$total~intC[inutt<=30]*inuttC[inutt<=30]+(1+intC[inutt<=30]*inuttC[in
coefs = data.frame(summary(lmo)$coefficients)
coefs$p = 2*(1-pnorm(abs(coefs$t.value)))
print(coefs)
```

```
##                                           Estimate Std..Error t.value       p
## (Intercept)                                 60.397      3.819 15.8146 0.00000
## intC[inutt <= 30]                           31.110     13.104  2.3740 0.01759
## inuttC[inutt <= 30]                         -2.899      3.107 -0.9331 0.35076
## intC[inutt <= 30]:inuttC[inutt <= 30]      20.581     11.335  1.8156 0.06943
```

```r
# debate 2
ggplot(debate[ixes,],aes(inutt,total,color=interrupt>0))+
    stat_summary(fun.data=mean_se,geom="pointrange",size=.5)+
    coord_cartesian(xlim=c(0,30),ylim=c(40,100))+
    scale_color_manual(values=colors)+
    scale_x_continuous(breaks=c(0,10,20,30,60))+
    xlab("")+ylab("")
```

```
## Warning: Removed 10 rows containing missing values (geom_segment).
## Warning: Removed 90 rows containing missing values (geom_segment).
```

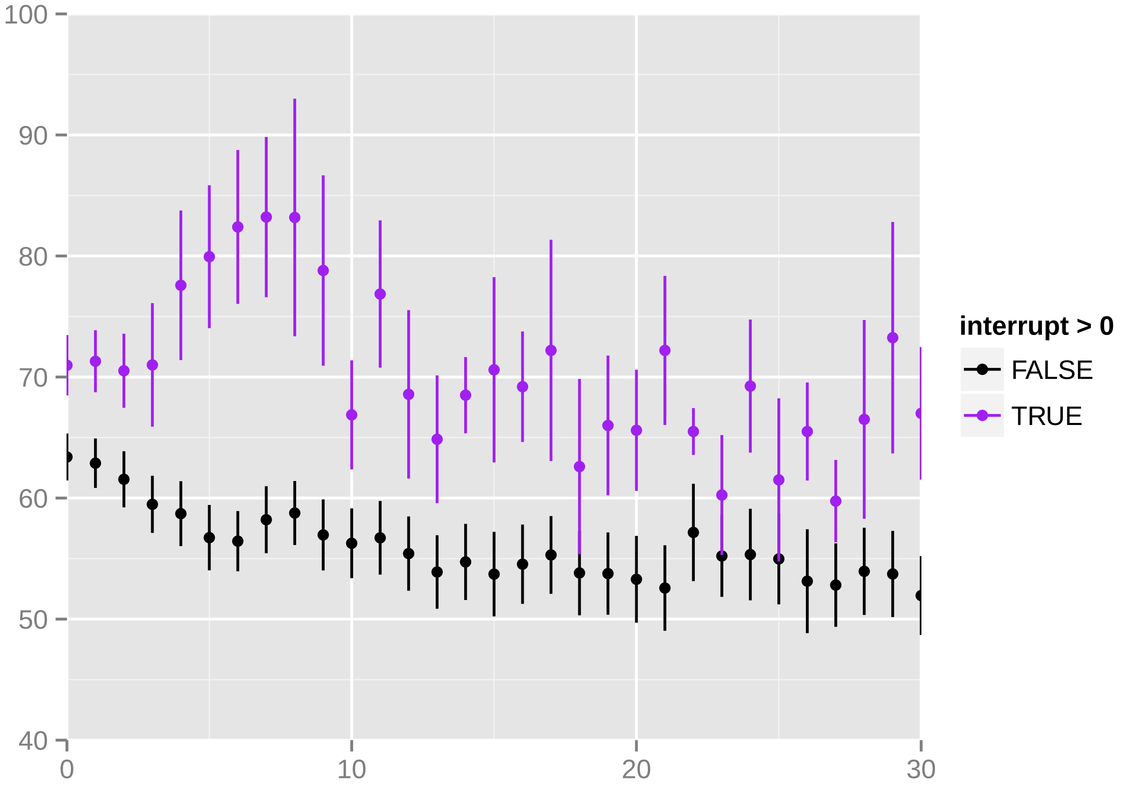


The following is an important set of information regarding the data coding and analysis.

Debate 1 starts in transcripts: 9:01:44.

Debate 2 starts in transcripts: 9:01:49.

Debate 3 starts in transcripts: 9:01:52

Timed using stopwatch + CSPAN clock – at the onset of speech.

From looking into the video / transcript:

big bird: 26:11

binders full of women 38:44

[horses and ]bayonets 42:03

For the time codes:

3-Interlocutor (1=Romney, 2=Obama, 3=Moderator, 4=Questioner in debate2)

6-Interruptions1 (1=interruption, 2=interrupting and taking the ground)

7-Interruptions2 (1=Moderator interrupts Romney, 2=M interrupts O, 3=O interrupts R, 4=R interrupts O, 5=R interrupts M, 6=O interrupts M)

# Influence on tweet rate by interruptions (Debate 3)

We load libraries and prepare parameters, as other *.Rmd's specify. This block also includes the alignment of data.

```
library(MuMIn)
library(lme4)
library(languageR)
library(ggplot2)

token_transcript_mention = c(26*60+11,38*60+44,42*60+3) # time when tokens (salient events) mentioned
transcripts_time_starts = c('Wed Oct  3 21:01:44 2012','Tue Oct 16 21:01:49 2012','Mon Oct 22 21:01:52 2

dnum = 3 # debate number

debate = read.table(paste('../data/debate',dnum,'.nort.txt',sep=''),head=TRUE,sep='\t') # load in debat
colnames(debate) = c('date','total','obama','romney','token')

### let's align the debate twitter data
align_index = which(debate$date==transcripts_time_starts[dnum]) # get row number where debate time star
debate = debate[align_index:nrow(debate),] # start debate data from second of debate onset

timecodes = read.table('../data/timecodes.txt',sep='\t',head=FALSE) # time codes from aarhus
colnames(timecodes) = c('startt','endt','who','speechturn','dnum','interrupt1','interrupt2','topic')
timecodes = timecodes[timecodes$dnum==dnum,] # take only the current debate

speakers = c('Romney','Obama','Moderator','Questioner (Debate 2)') # just for reference (see notes)
colors = c('black','purple')
```

In the next block, we determine the intervals which involve any form of interruption. Get some descriptives, too.

```
dim(timecodes)[1]
```

```
## [1] 190
```

```
sum(timecodes$interrupt1>0)
```

```
## [1] 117
```

```
ints = timecodes[timecodes$interrupt1>0,]
min(ints$endt-ints$startt) # shortest interruption
```

```
## [1] 0.1
```

```
max(ints$endt-ints$startt) # longest interruption
```

```
## [1] 117.7
```

```r
table(ints$who) # who interrupts whom here?
```

```
##
##  1  2  3
## 45 41 31
```

Next we use these intervals to create predictors based on the sequence of interruptions (second-by-second).

```r
# indices included in the analysis (reflecting # of seconds into
# debate, with debate array starting at 0, so can index using row #)
ixes = c()  # seconds / rows to be used to inspect twitter data (seconds can reference rows, since deba
inutt = c() # the time that the turn has been going on
interrupt = c() # whether a turn is an interruption
speechturn = c() # unique segment #, as random factor, to ensure not driven by small # of utterances

timecodes$endt = round(timecodes$endt) # can be used to reference debate directly, need integers
timecodes$startt = round(timecodes$startt)

for (j in 2:nrow(timecodes)) { # moderator always 1, skip
    ixes = c(ixes,(timecodes[j,]$startt:timecodes[j,]$endt)) # get range of s
    inutt = c(inutt,(timecodes[j,]$startt:timecodes[j,]$endt)-timecodes[j,]$startt)
    speechturn = c(speechturn,(timecodes[j,]$startt:timecodes[j,]$endt)*0+timecodes[j,]$speechturn)
    # is this an interruption
    interrupt = c(interrupt,(timecodes[j,]$startt:timecodes[j,]$endt)*0+(timecodes[j,]$interrupt1>0)*1)
}
```

Now that we have our predictors we can run full lmer models to determine whether interruptions contribute to tweet rate. We then plot.

```r
# check then use fully saturated (maximal) random effect structure within speech turns
intC = interrupt # keep it dichotomous; results consistent, just change utt coefficient (uninteresting)
inuttC = (inutt - mean(inutt))/sd(inutt)
dv = (debate[ixes,]$total - mean(debate[ixes,]$total))/sd(debate[ixes,]$total)
lmo = lmer(dv~intC*inuttC+(1+intC*inuttC|speechturn),data=debate[ixes,])
```

```
## Warning: Model failed to converge: degenerate Hessian with 1 negative
## eigenvalues
```

```r
coefs = data.frame(summary(lmo)$coefficients)
coefs$p = 2*(1-pnorm(abs(coefs$t.value)))
print(coefs)
```

```
##             Estimate Std..Error t.value         p
## (Intercept) -0.09627    0.08983  -1.072 2.838e-01
## intC         0.93723    0.22519   4.162 3.154e-05
## inuttC      -0.07734    0.05757  -1.343 1.791e-01
## intC:inuttC  0.29063    0.17624   1.649 9.915e-02
```

```r
r.squaredGLMM(lmo)
```

```
##    R2m    R2c
## 0.1200 0.7061
```

```
# check for first 30 seconds (consistent)
lmo = lmer(debate[ixes[inutt<=30],]$total~intC[inutt<=30]*inuttC[inutt<=30]+(1+intC[inutt<=30]*inuttC[in
```

```
## Warning: Model is nearly unidentifiable: large eigenvalue ratio
##  - Rescale variables?
```

```
coefs = data.frame(summary(lmo)$coefficients)
coefs$p = 2*(1-pnorm(abs(coefs$t.value)))
print(coefs)
```

```
##                                        Estimate Std..Error t.value
## (Intercept)                              32.932      1.378  23.906
## intC[inutt <= 30]                         9.778      2.499   3.914
## inuttC[inutt <= 30]                      -1.332      1.114  -1.196
## intC[inutt <= 30]:inuttC[inutt <= 30]     2.804      1.958   1.432
##                                                 p
## (Intercept)                             0.000e+00
## intC[inutt <= 30]                       9.095e-05
## inuttC[inutt <= 30]                     2.318e-01
## intC[inutt <= 30]:inuttC[inutt <= 30] 1.522e-01
```

```
# debate 3
ggplot(debate[ixes,],aes(inutt,total,color=interrupt>0))+
    stat_summary(fun.data=mean_se,geom="pointrange",size=.5)+
    coord_cartesian(xlim=c(0,30),ylim=c(25,50))+
    scale_color_manual(values=colors)+
    scale_x_continuous(breaks=c(0,10,20,30,40,50,60))+
    scale_y_continuous(breaks=c(25,30,35,40,45,50))+
    xlab("")+ylab("")
```

```
## Warning: Removed 4 rows containing missing values (geom_segment).
## Warning: Removed 15 rows containing missing values (geom_segment).
```

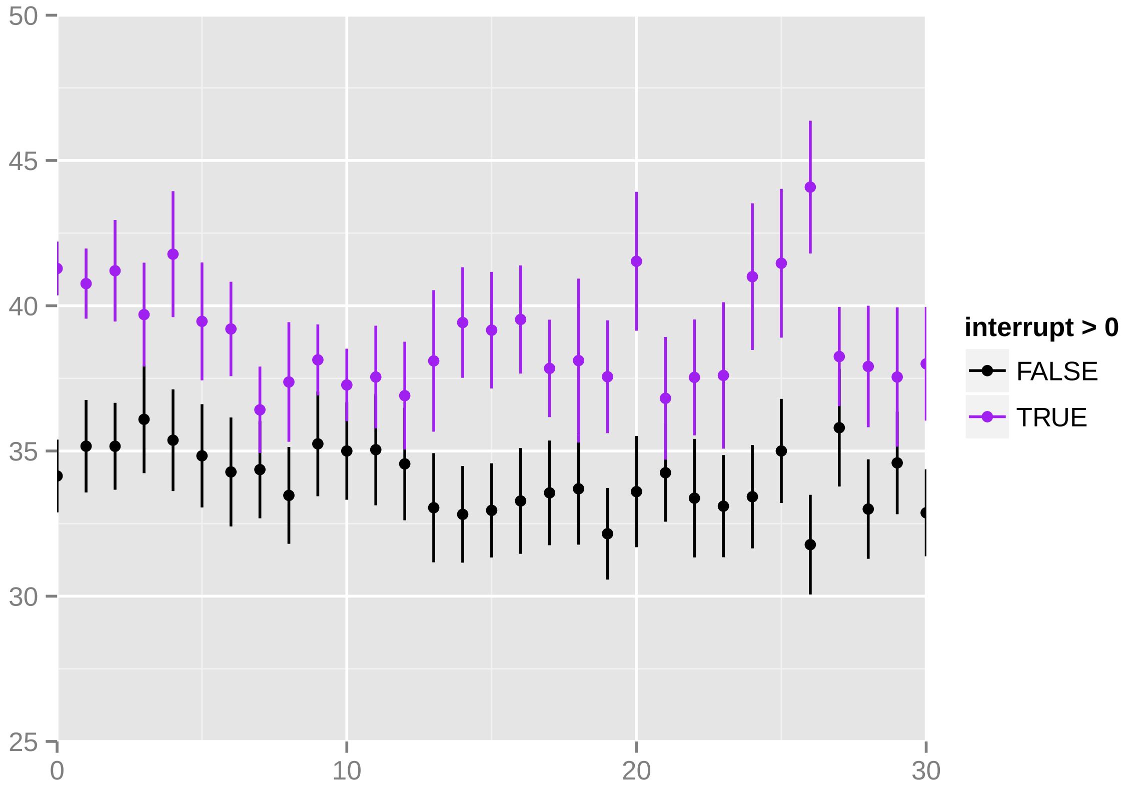


The following is an important set of information regarding the data coding and analysis.

Debate 1 starts in transcripts: 9:01:44.

Debate 2 starts in transcripts: 9:01:49.

Debate 3 starts in transcripts: 9:01:52

Timed using stopwatch + CSPAN clock – at the onset of speech.

From looking into the video / transcript:

big bird: 26:11

binders full of women 38:44

[horses and ]bayonets 42:03

For the time codes:

3-Interlocutor (1=Romney, 2=Obama, 3=Moderator, 4=Questioner in debate2)

6-Interruptions1 (1=interruption, 2=interrupting and taking the ground)

7-Interruptions2 (1=Moderator interrupts Romney, 2=M interrupts O, 3=O interrupts R, 4=R interrupts O, 5=R interrupts M, 6=O interrupts M)

# Plot of the salient events in each debate

See other markdowns for explanation here.

```
token_transcript_mention = c(26*60+11,38*60+44,42*60+3)
transcripts_time_starts = c('Wed Oct  3 21:01:44 2012','Tue Oct 16 21:01:49 2012','Mon Oct 22 21:01:52 2
```

```
trans = .5
cols = c(rgb(.5,0,.5,trans),rgb(0,.5,.5,trans),rgb(.5,.5,0,trans)) # simply colors each token/salient e
cols2 = c(rgb(.5,0,.5,1),rgb(0,.5,.5,1),rgb(.5,.5,0,1))

#dev.new(width=3, height=4)
for (dnum in 1:3) {
    debate = read.table(paste('../data/debate',dnum,'.nort.txt',sep=''),head=TRUE,sep='\t')
    debateRT = read.table(paste('../data/debate',dnum,'.txt',sep=''),head=TRUE,sep='\t')

    colnames(debate) = c('date','total','obama','romney','token')
    colnames(debateRT) = c('date','total','obama','romney','token')

    align_index = which(debate$date==transcripts_time_starts[dnum]) ### let's align the debate twitter d
    align_indexRT = which(debateRT$date==transcripts_time_starts[dnum]) ### let's align the debate twit

    debate = debate[align_index:nrow(debate),]
    debateRT = debateRT[align_indexRT:nrow(debateRT),]

  # grab the token for this debate from -100 before it appears up to 600 seconds after
    dtRT = debateRT[(token_transcript_mention[dnum]-100):(token_transcript_mention[dnum]+600),]$token
    dt = debate[(token_transcript_mention[dnum]-100):(token_transcript_mention[dnum]+600),]$token

    mx = max(debate[(token_transcript_mention[dnum]-100):(token_transcript_mention[dnum]+600),]$token)

    mxRT = max(dtRT - dt)        # get retweets minus non-retweets (diff = retweets left over)
    dtRT = dtRT - dt    # get retweets minus non-retweets (diff = retweets left over)

    print(which.max(dtRT)-100)
    print(which(dtRT==1)[1]-100)

    #### plot max scaled; remove RT for no retweets
    if (dnum==1) plot(-100:600,dtRT / mxRT, col=cols[dnum],xlab=' ',ylab=' ',ann=FALSE,pch=19,cex=.5,xa
    else points(-100:600,dtRT / mxRT, col=cols[dnum],pch=19,cex=.5)

    #ts = poly(-100:600,20)
    #lmo = glm(dt~ts[,1:20],family=poisson)
    #points(predict(lmo)/mx,type='l',col=cols2[dnum],lw=2)
    #lines(spline(dt/mx,n=10),col='red')

}
```

```
## [1] 99
## [1] 31

## [1] 80
```

```
## [1] 14
## [1] 78
## [1] 17
```

```
axis(1,at=c(0,200,400,600),las=1,cex.axis=.75,mgp=c(1, .3, 0))
mtext(side = 1, text = " ", line = 1,cex=.75)
mtext(side = 2, text = " ", line = 1.25,cex=.75)
legend("topright",c('Big Bird','binder','bayonet'),col=cols2,pch=19,bty="n",cex=.7)
```

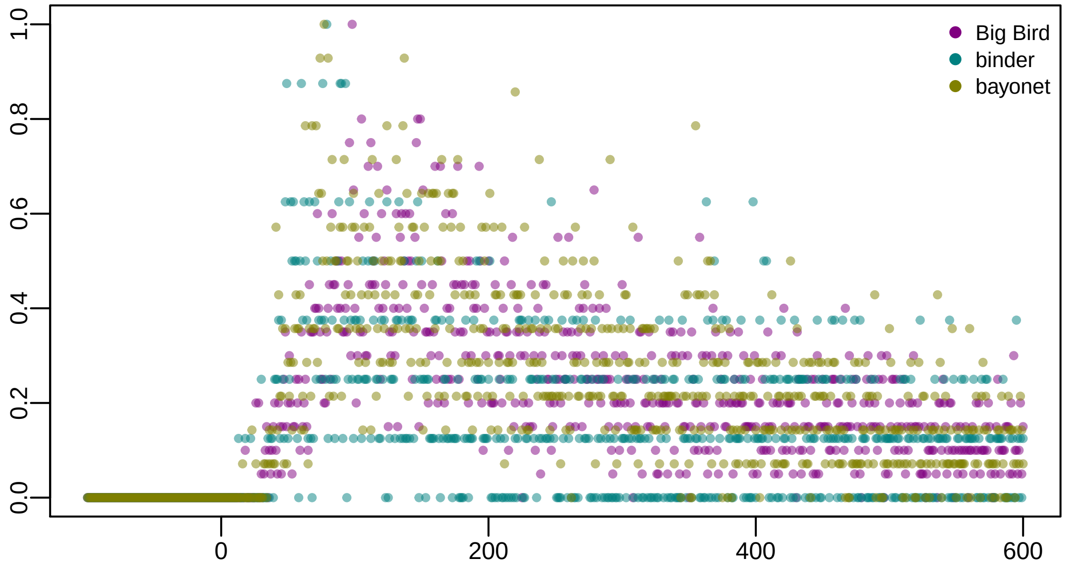

# Simple model of a meme, with parameter search fit and plot (Debate 1)

Same as previous markdowns for getting salient event time points.

```
token_transcript_mention = c(26*60+11,38*60+44,42*60+3)
transcripts_time_starts = c('Wed Oct  3 21:01:44 2012','Tue Oct 16 21:01:49 2012','Mon Oct 22 21:01:52 2

dnum = 1 # debate number

debate = read.table(paste('../data/debate',dnum,'.nort.txt',sep=''),head=TRUE,sep='\t')
debateRT = read.table(paste('../data/debate',dnum,'.txt',sep=''),head=TRUE,sep='\t')
colnames(debate) = c('date','total','obama','romney','token')
colnames(debateRT) = c('date','total','obama','romney','token')

align_index = which(debate$date==transcripts_time_starts[dnum]) ### let's align the debate twitter data
debate = debate[align_index:nrow(debate),]

timecodes = read.table('../data/timecodes.txt',sep='\t',head=FALSE)
colnames(timecodes) = c('startt','endt','who','speechturn','dnum','interrupt1','interrupt2','topic')
timecodes = timecodes[timecodes$dnum==dnum,]

speakers = c('Romney','Obama','Moderator','Questioner (Debate 2)')
colors = c('red','blue')


# plot the stone in the pond (tokens)
token_transcript_mention = c(26*60+11,38*60+44,42*60+3)
transcripts_time_starts = c('Wed Oct  3 21:01:44 2012','Tue Oct 16 21:01:49 2012','Mon Oct 22 21:01:52 2

trans = .5
cols = c(rgb(.5,0,.5,trans),rgb(0,.5,.5,trans),rgb(.5,.5,0,trans))
cols2 = c(rgb(.5,0,.5,1),rgb(0,.5,.5,1),rgb(.5,.5,0,1))
#dev.new(width=3, height=4)
for (dnum in 1) {
    debate = read.table(paste('../data/debate',dnum,'.nort.txt',sep=''),head=TRUE,sep='\t')
    debateRT = read.table(paste('../data/debate',dnum,'.txt',sep=''),head=TRUE,sep='\t')

    colnames(debate) = c('date','total','obama','romney','token')
    colnames(debateRT) = c('date','total','obama','romney','token')

    align_index = which(debate$date==transcripts_time_starts[dnum]) ### let's align the debate twitter d
    align_indexRT = which(debateRT$date==transcripts_time_starts[dnum]) ### let's align the debate twit

    debate = debate[align_index:nrow(debate),]
    debateRT = debateRT[align_indexRT:nrow(debateRT),]

    dtRT = debateRT[(token_transcript_mention[dnum]-100):(token_transcript_mention[dnum]+600),]$token
    dt = debate[(token_transcript_mention[dnum]-100):(token_transcript_mention[dnum]+600),]$token

    mx = max(debate[(token_transcript_mention[dnum]-100):(token_transcript_mention[dnum]+600),]$token)
```

```
    mxRT = max(dtRT - dt)       # get retweets minus non-retweets (diff = retweets left over)
    dtRT = dtRT - dt   # get retweets minus non-retweets (diff = retweets left over)

    print(which.max(dtRT)-100)
    print(which(dtRT==1)[1]-100)

    #### plot max scaled
    plot(-100:600,dt / mx, col=rgb(.7,.7,.7),xlab=' ',ylab=' ',ann=FALSE,pch=19,cex=.5,xaxt='n',cex.axi
    #else points(-100:600,dt / mx, col=rgb(.7,.7,.7),pch=19,cex=.5)

    #ts = poly(-100:600,20)
    #lmo = glm(dt~ts[,1:20],family=poisson)
    #points(predict(lmo)/mx,type='l',col=cols2[dnum],lw=2)
    #lines(spline(dt/mx,n=10),col='red')

}
```

```
## [1] 99
## [1] 31
```

```
axis(1,at=c(0,200,400,600),las=1,cex.axis=.75,mgp=c(1, .3, 0))
mtext(side = 1, text = " ", line = 1,cex=.75)
mtext(side = 2, text = " ", line = 1.25,cex=.75)
#legend("topright",c('Big Bird','binder','bayonet'),col=cols2,pch=19,bty="n",cex=.7)

t = 0:600
shift1 = round((which.max(dt)-100)/2)
base_rate = mean(dt[600:700])/mx

library(matlab)

corrs = c()
shifts = c()
lambs = c()
divs = c()
ks = c()
for (k in 1:10) {
    # solving for slope that achieves max value at some x value for sigmoid
    slope1 = -1 * ( (log(.1)+log(.9)) / ((which.max(dt)-100)/k) )
    for (j in linspace(0,300,21)) {
        s1 = (1/(1+exp(-(slope1*(t-j))))) # shifting sigmoid
        for (i in linspace(0,360,41)) {
            lamb = -log(base_rate) / i # finding lambda at which the tweet rate bends
            dt_pred = exp(-lamb*t)*s1+base_rate*s1
            corrs = c(corrs,cor.test(dt_pred,dt[100:700])$estimate)
            lambs = c(lambs,i)
            shifts = c(shifts,j)
      ks = c(ks,k)
        }
    }
}
#plot(corrs)
```

```
lamb = -log(base_rate) / lambs[which.max(corrs)] # finding lambda at which the tweet rate bends
shift1 = shifts[which.max(corrs)]
k = ks[which.max(corrs)]

# note -100 because we are starting at 0 not -100 (the tweets for salient events shows -100 before, whi
slope1 = -1 * ( (log(.1)+log(.9)) / ((which.max(dt)-100)/k) ) # solving for slope that achieves max val
s1 = (1/(1+exp(-(slope1*(t-shift1))))) # shifting sigmoid

points(t,base_rate*s1,'type'='l')
points(exp(-lamb*t),type='l')
points(exp(-lamb*t)*s1+base_rate*s1,type='l',lwd=2)
```

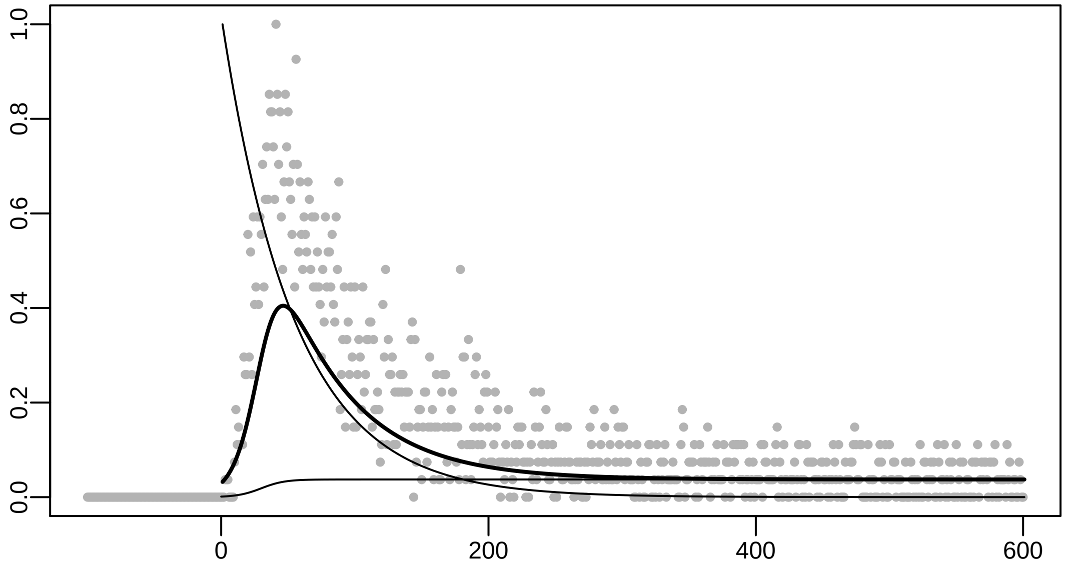


```
# decay*sigmoid + simmer*sigmoid
```

# Simple model of a meme, with parameter search fit and plot (Debate 2)

Same as previous markdowns for getting salient event time points.

```
token_transcript_mention = c(26*60+11,38*60+44,42*60+3)
transcripts_time_starts = c('Wed Oct  3 21:01:44 2012','Tue Oct 16 21:01:49 2012','Mon Oct 22 21:01:52 2

dnum = 2 # debate number

debate = read.table(paste('../data/debate',dnum,'.nort.txt',sep=''),head=TRUE,sep='\t')
debateRT = read.table(paste('../data/debate',dnum,'.txt',sep=''),head=TRUE,sep='\t')
colnames(debate) = c('date','total','obama','romney','token')
colnames(debateRT) = c('date','total','obama','romney','token')

align_index = which(debate$date==transcripts_time_starts[dnum]) ### let's align the debate twitter data
debate = debate[align_index:nrow(debate),]

timecodes = read.table('../data/timecodes.txt',sep='\t',head=FALSE)
colnames(timecodes) = c('startt','endt','who','speechturn','dnum','interrupt1','interrupt2','topic')
timecodes = timecodes[timecodes$dnum==dnum,]

speakers = c('Romney','Obama','Moderator','Questioner (Debate 2)')
colors = c('red','blue')


# plot the stone in the pond (tokens)
token_transcript_mention = c(26*60+11,38*60+44,42*60+3)
transcripts_time_starts = c('Wed Oct  3 21:01:44 2012','Tue Oct 16 21:01:49 2012','Mon Oct 22 21:01:52 2

trans = .5
cols = c(rgb(.5,0,.5,trans),rgb(0,.5,.5,trans),rgb(.5,.5,0,trans))
cols2 = c(rgb(.5,0,.5,1),rgb(0,.5,.5,1),rgb(.5,.5,0,1))
#dev.new(width=3, height=4)
for (dnum in 2) {
    debate = read.table(paste('../data/debate',dnum,'.nort.txt',sep=''),head=TRUE,sep='\t')
    debateRT = read.table(paste('../data/debate',dnum,'.txt',sep=''),head=TRUE,sep='\t')

    colnames(debate) = c('date','total','obama','romney','token')
    colnames(debateRT) = c('date','total','obama','romney','token')

    align_index = which(debate$date==transcripts_time_starts[dnum]) ### let's align the debate twitter d
    align_indexRT = which(debateRT$date==transcripts_time_starts[dnum]) ### let's align the debate twit

    debate = debate[align_index:nrow(debate),]
    debateRT = debateRT[align_indexRT:nrow(debateRT),]

    dtRT = debateRT[(token_transcript_mention[dnum]-100):(token_transcript_mention[dnum]+600),]$token
    dt = debate[(token_transcript_mention[dnum]-100):(token_transcript_mention[dnum]+600),]$token

    mx = max(debate[(token_transcript_mention[dnum]-100):(token_transcript_mention[dnum]+600),]$token)
```

```r
    mxRT = max(dtRT - dt)        # get retweets minus non-retweets (diff = retweets left over)
    dtRT = dtRT - dt    # get retweets minus non-retweets (diff = retweets left over)

    print(which.max(dtRT)-100)
    print(which(dtRT==1)[1]-100)

    #### plot max scaled
    plot(-100:600,dt / mx, col=rgb(.7,.7,.7),xlab=' ',ylab=' ',ann=FALSE,pch=19,cex=.5,xaxt='n',cex.axi
    #else points(-100:600,dt / mx, col=rgb(.7,.7,.7),pch=19,cex=.5)

    #ts = poly(-100:600,20)
    #lmo = glm(dt~ts[,1:20],family=poisson)
    #points(predict(lmo)/mx,type='l',col=cols2[dnum],lw=2)
    #lines(spline(dt/mx,n=10),col='red')

}
```

```
## [1] 80
## [1] 14
```

```r
axis(1,at=c(0,200,400,600),las=1,cex.axis=.75,mgp=c(1, .3, 0))
mtext(side = 1, text = " ", line = 1,cex=.75)
mtext(side = 2, text = " ", line = 1.25,cex=.75)
#legend("topright",c('Big Bird','binder','bayonet'),col=cols2,pch=19,bty="n",cex=.7)

t = 0:600
shift1 = round((which.max(dt)-100)/2)
base_rate = mean(dt[600:700])/mx

library(matlab)

corrs = c()
shifts = c()
lambs = c()
divs = c()
ks = c()
for (k in 1:10) {
    # solving for slope that achieves max value at some x value for sigmoid
    slope1 = -1 * ( (log(.1)+log(.9)) / ((which.max(dt)-100)/k) )
    for (j in linspace(0,300,21)) {
        s1 = (1/(1+exp(-(slope1*(t-j))))) # shifting sigmoid
        for (i in linspace(0,360,41)) {
            lamb = -log(base_rate) / i # finding lambda at which the tweet rate bends
            dt_pred = exp(-lamb*t)*s1+base_rate*s1
            corrs = c(corrs,cor.test(dt_pred,dt[100:700])$estimate)
            lambs = c(lambs,i)
            shifts = c(shifts,j)
      ks = c(ks,k)
        }
    }
}
#plot(corrs)
```

```
lamb = -log(base_rate) / lambs[which.max(corrs)] # finding lambda at which the tweet rate bends
shift1 = shifts[which.max(corrs)]
k = ks[which.max(corrs)]

# note -100 because we are starting at 0 not -100 (the tweets for salient events shows -100 before, whi
slope1 = -1 * ( (log(.1)+log(.9)) / ((which.max(dt)-100)/k) ) # solving for slope that achieves max val
s1 = (1/(1+exp(-(slope1*(t-shift1))))) # shifting sigmoid

points(t,base_rate*s1,'type'='l')
points(exp(-lamb*t),type='l')
points(exp(-lamb*t)*s1+base_rate*s1,type='l',lwd=2)
```

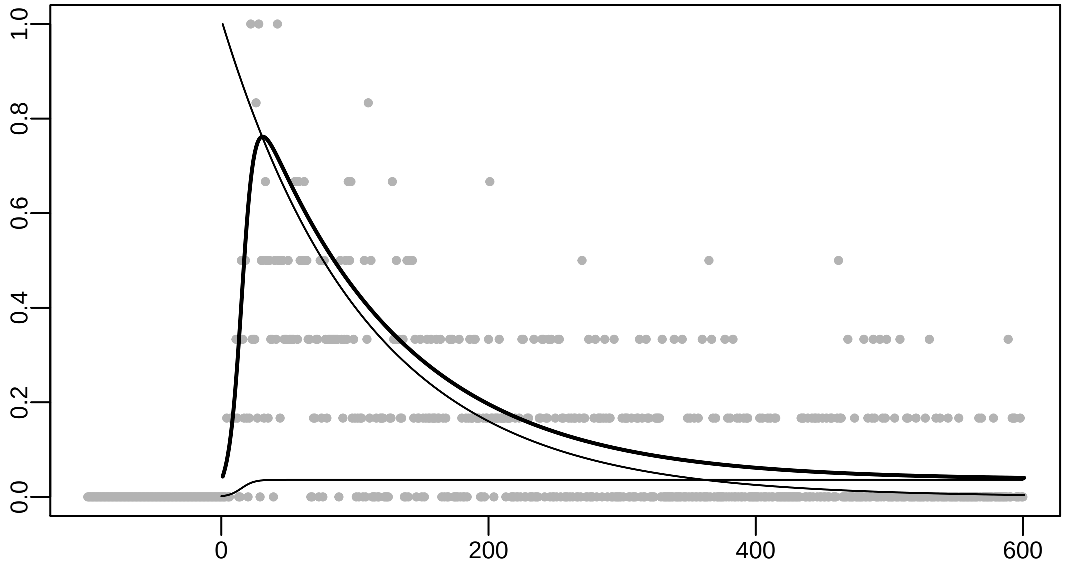


```
# decay*sigmoid + simmer*sigmoid
```

# Simple model of a meme, with parameter search fit and plot (Debate 3)

Same as previous markdowns for getting salient event time points.

```
token_transcript_mention = c(26*60+11,38*60+44,42*60+3)
transcripts_time_starts = c('Wed Oct  3 21:01:44 2012','Tue Oct 16 21:01:49 2012','Mon Oct 22 21:01:52 

dnum = 3 # debate number

debate = read.table(paste('../data/debate',dnum,'.nort.txt',sep=''),head=TRUE,sep='\t')
debateRT = read.table(paste('../data/debate',dnum,'.txt',sep=''),head=TRUE,sep='\t')
colnames(debate) = c('date','total','obama','romney','token')
colnames(debateRT) = c('date','total','obama','romney','token')

align_index = which(debate$date==transcripts_time_starts[dnum]) ### let's align the debate twitter data
debate = debate[align_index:nrow(debate),]

timecodes = read.table('../data/timecodes.txt',sep='\t',head=FALSE)
colnames(timecodes) = c('startt','endt','who','speechturn','dnum','interrupt1','interrupt2','topic')
timecodes = timecodes[timecodes$dnum==dnum,]

speakers = c('Romney','Obama','Moderator','Questioner (Debate 2)')
colors = c('red','blue')


# plot the stone in the pond (tokens)
token_transcript_mention = c(26*60+11,38*60+44,42*60+3)
transcripts_time_starts = c('Wed Oct  3 21:01:44 2012','Tue Oct 16 21:01:49 2012','Mon Oct 22 21:01:52 

trans = .5
cols = c(rgb(.5,0,.5,trans),rgb(0,.5,.5,trans),rgb(.5,.5,0,trans))
cols2 = c(rgb(.5,0,.5,1),rgb(0,.5,.5,1),rgb(.5,.5,0,1))
#dev.new(width=3, height=4)
for (dnum in 3) {
    debate = read.table(paste('../data/debate',dnum,'.nort.txt',sep=''),head=TRUE,sep='\t')
    debateRT = read.table(paste('../data/debate',dnum,'.txt',sep=''),head=TRUE,sep='\t')

    colnames(debate) = c('date','total','obama','romney','token')
    colnames(debateRT) = c('date','total','obama','romney','token')

    align_index = which(debate$date==transcripts_time_starts[dnum]) ### let's align the debate twitter 
    align_indexRT = which(debateRT$date==transcripts_time_starts[dnum]) ### let's align the debate twit

    debate = debate[align_index:nrow(debate),]
    debateRT = debateRT[align_indexRT:nrow(debateRT),]

    dtRT = debateRT[(token_transcript_mention[dnum]-100):(token_transcript_mention[dnum]+600),]$token
    dt = debate[(token_transcript_mention[dnum]-100):(token_transcript_mention[dnum]+600),]$token

    mx = max(debate[(token_transcript_mention[dnum]-100):(token_transcript_mention[dnum]+600),]$token)
```

```r
    mxRT = max(dtRT - dt)       # get retweets minus non-retweets (diff = retweets left over)
    dtRT = dtRT - dt    # get retweets minus non-retweets (diff = retweets left over)

    print(which.max(dtRT)-100)
    print(which(dtRT==1)[1]-100)

    #### plot max scaled
    plot(-100:600,dt / mx, col=rgb(.7,.7,.7),xlab=' ',ylab=' ',ann=FALSE,pch=19,cex=.5,xaxt='n',cex.axi
    #else points(-100:600,dt / mx, col=rgb(.7,.7,.7),pch=19,cex=.5)

    #ts = poly(-100:600,20)
    #lmo = glm(dt~ts[,1:20],family=poisson)
    #points(predict(lmo)/mx,type='l',col=cols2[dnum],lw=2)
    #lines(spline(dt/mx,n=10),col='red')

}
```

```
## [1] 78
## [1] 17
```

```r
axis(1,at=c(0,200,400,600),las=1,cex.axis=.75,mgp=c(1, .3, 0))
mtext(side = 1, text = " ", line = 1,cex=.75)
mtext(side = 2, text = " ", line = 1.25,cex=.75)
#legend("topright",c('Big Bird','binder','bayonet'),col=cols2,pch=19,bty="n",cex=.7)

t = 0:600
shift1 = round((which.max(dt)-100)/2)
base_rate = mean(dt[600:700])/mx

library(matlab)

corrs = c()
shifts = c()
lambs = c()
divs = c()
ks = c()
for (k in 1:10) {
    # solving for slope that achieves max value at some x value for sigmoid
    slope1 = -1 * ( (log(.1)+log(.9)) / ((which.max(dt)-100)/k) )
    for (j in linspace(0,300,21)) {
        s1 = (1/(1+exp(-(slope1*(t-j))))) # shifting sigmoid
        for (i in linspace(0,360,41)) {
            lamb = -log(base_rate) / i # finding lambda at which the tweet rate bends
            dt_pred = exp(-lamb*t)*s1+base_rate*s1
            corrs = c(corrs,cor.test(dt_pred,dt[100:700])$estimate)
            lambs = c(lambs,i)
            shifts = c(shifts,j)
      ks = c(ks,k)
        }
    }
}
#plot(corrs)
```

```
lamb = -log(base_rate) / lambs[which.max(corrs)] # finding lambda at which the tweet rate bends
shift1 = shifts[which.max(corrs)]
k = ks[which.max(corrs)]

# note -100 because we are starting at 0 not -100 (the tweets for salient events shows -100 before, whi
slope1 = -1 * ( (log(.1)+log(.9)) / ((which.max(dt)-100)/k) ) # solving for slope that achieves max val
s1 = (1/(1+exp(-(slope1*(t-shift1))))) # shifting sigmoid

points(t,base_rate*s1,'type'='l')
points(exp(-lamb*t),type='l')
points(exp(-lamb*t)*s1+base_rate*s1,type='l',lwd=2)
```

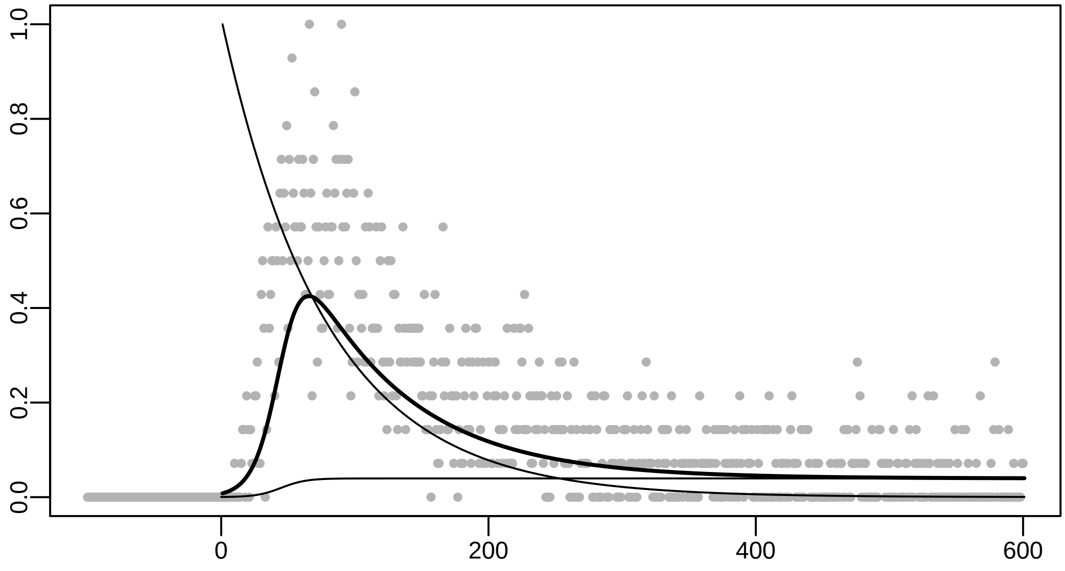


```
# decay*sigmoid + simmer*sigmoid
```

# A breakdown of the regression model and some plots (Debate 1)

```
setwd('/Users/rickdale/Dropbox/projects/papers/presidentialdebates_full/final_analysis/markdowns/Figure
token_transcript_mention = c(26*60+11,38*60+44,42*60+3) ### the by-the-second occurrence of the meme eve
transcripts_time_starts = c('Wed Oct  3 21:01:44 2012','Tue Oct 16 21:01:49 2012','Mon Oct 22 21:01:52 

dnum = 1 # debate number

debate = read.table(paste('../data/debate',dnum,'.nort.txt',sep=''),head=TRUE,sep='\t') ### get the twi
colnames(debate) = c('date','total','obama','romney','token')

align_index = which(debate$date==transcripts_time_starts[dnum]) ### let's align the debate twitter data
debate = debate[align_index:nrow(debate),] ### start from where the transcript begins (timelocking)

timecodes = read.table('../data/timecodes.txt',sep='\t',head=FALSE) ### get the transcript event segmen
colnames(timecodes) = c('startt','endt','who','speechturn','dnum','interrupt1','interrupt2','topic')
timecodes = timecodes[timecodes$dnum==dnum,] ### only get the current debate for analysis

speakers = c('Romney','Obama','Moderator','Questioner (Debate 2)')
colors = c('red','blue')
```

This function finds peaks in the twitter data, indicating potential "salient events" models them simply as a slow decay; varies from 0 to 1.

```
pulses = function(tweets) {
  tweets_sm = supsmu(1:length(tweets),tweets,span=.01);
    diffs = diff(tweets_sm$y,2)
    spliney = spline(1:length(tweets),tweets,n=50)
    diffs = diff(spliney$y,1)
    change_dirs = (diffs[2:length(diffs)]<0 & diffs[1:length(diffs)-1]>0)
    indices = which(change_dirs==T)+1
    tweets_peaks = round(spliney$x[indices])
    ps = c()
    for (p in 1:length(tweets_peaks)) {
    ln = length(tweets)-tweets_peaks[p]
    rg = 1:ln
    tail = (rg)
    tail = tail^(-.5)
    psc = c(0*(1:(tweets_peaks[p])),tail)
    ps = cbind(ps,psc)
    }
    pulses = ps
}
```

Create the time series to be used in the regression model.

```
temp = timecodes
temp$who2 = temp$who # who is talking
temp$startt = round(temp$startt) # take it /second
temp$endt = round(temp$endt)    # take it /second
```

```
ixes = c() # indices of alignment between twitter and speech (in seconds)
speechturn = c()
who = c()
whoix1 = c();whoix2 = c()
interrupt = c()
for (j in 1:nrow(temp)) {
  ixes = c(ixes,temp[j,]$startt:temp[j,]$endt)
  speechturn = c(speechturn,(temp[j,]$startt:temp[j,]$endt)*0+temp[j,]$speechturn)
  who = c(who,(temp[j,]$startt:temp[j,]$endt)*0+temp[j,]$who2)
  whoix1 = c(whoix1,1*(temp[j,]$who2==1)*c(1:length(temp[j,]$startt:temp[j,]$endt))) # romney talking i
  whoix2 = c(whoix2,1*(temp[j,]$who2==2)*c(1:length(temp[j,]$startt:temp[j,]$endt))) # obama talking in
  interrupt = c(interrupt,(temp[j,]$startt:temp[j,]$endt)*0+temp[j,]$interrupt1)
}
# since ixes is /second, and will refer to tweet data, remove any 0 that may be at the beginning
if (ixes[1]==0) {
    ixes=ixes[2:length(ixes)];
    who=who[2:length(who)];
    speechturn=speechturn[2:length(speechturn)]
    whoix1 = whoix1[2:length(whoix1)]
    whoix2 = whoix2[2:length(whoix2)]
    interrupt = interrupt[2:length(interrupt)]
}
qtime = poly(1:length(who),2) # rise/fall of the debate
event = c(0*(1:(token_transcript_mention[1]-1)),(token_transcript_mention[1]/(token_transcript_mention[
twitter_data = debate[ixes,] # just get the indices from the twitter data from time 0 to how much of th
ps = pulses(twitter_data$total)
# let's center
whoix2C=(whoix2-mean(whoix2));whoix1C=(whoix1-mean(whoix1));interruptC=interrupt-mean(interrupt);
```

The following code plots the predictors to gain a clear sense of how they will be capturing the variance of the tweet rate for this debate.

```
#dev.new(width=6, height=3)
plot(-1*qtime[,2],type='l',cex=.1,xlab='',ylab='',xaxt='none',yaxt='none') # quadratic time term ("long
points(qtime[,1],type='l',cex=.1)
```

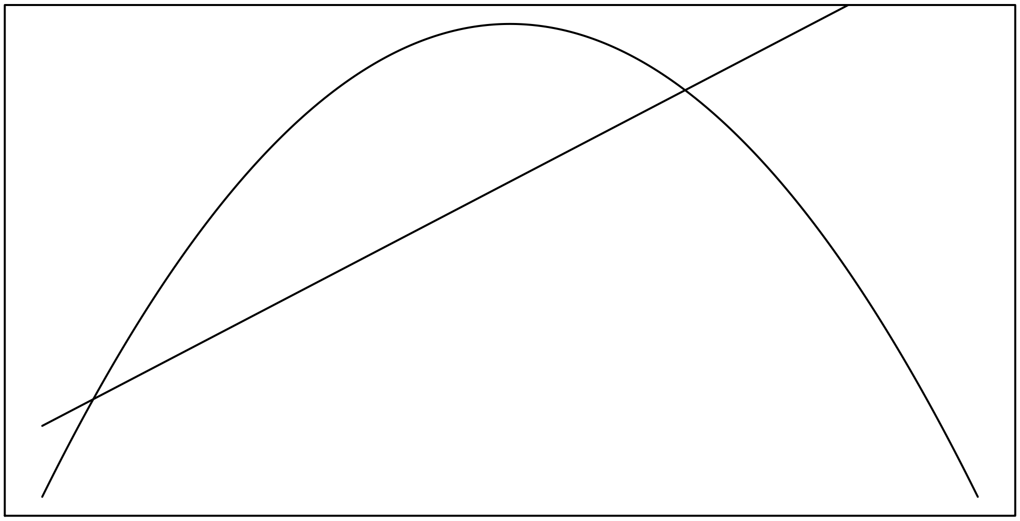

```
plot(whoix1,type='p',col='red',cex=.1,xlab='',ylab='',xaxt='none',yaxt='none') # romney
```

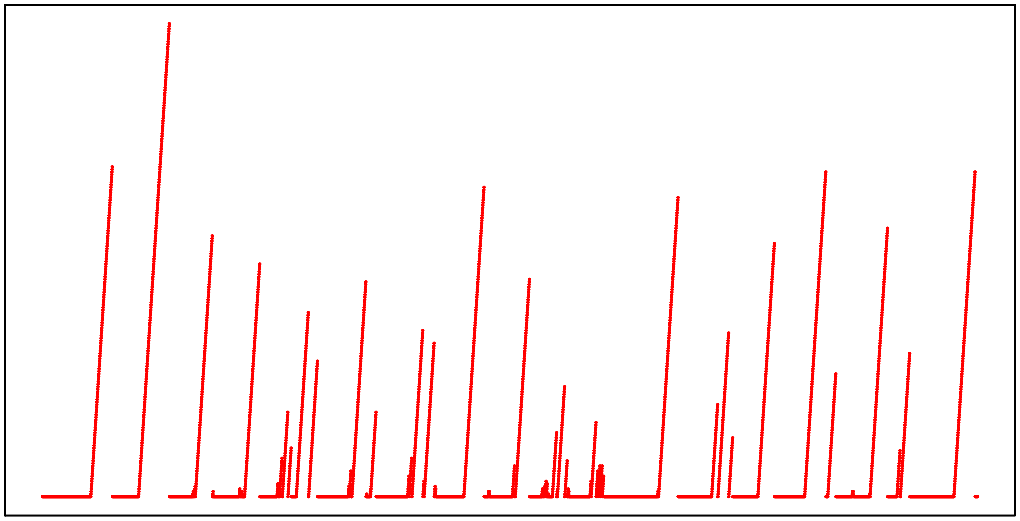

```
plot(whoix2,type='p',col='blue',cex=.1,xlab='',ylab='',xaxt='none',yaxt='none') # obama
```

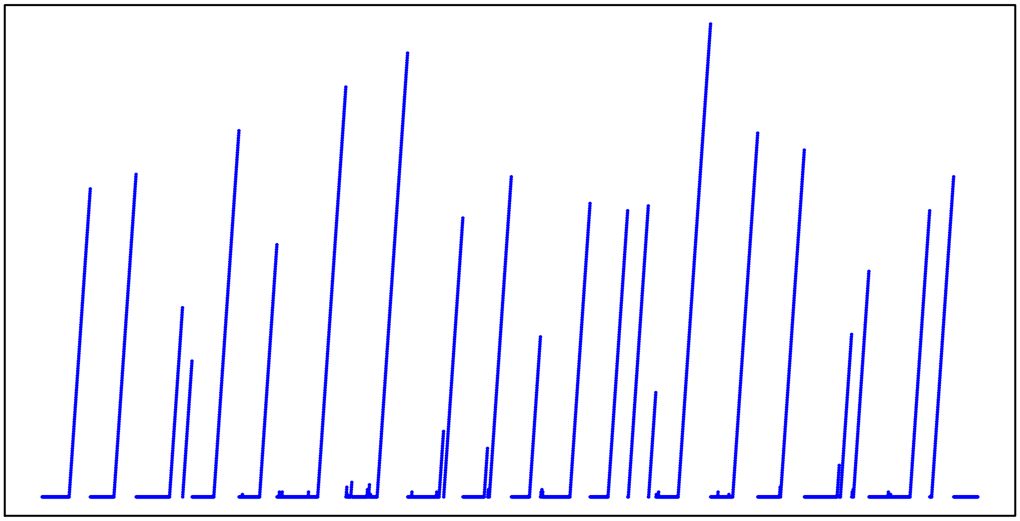

```
plot(interrupt>0,type='l',cex=.1,ylab='',yaxt='none',xlab='',xaxt='none') # was it an interruption?
```

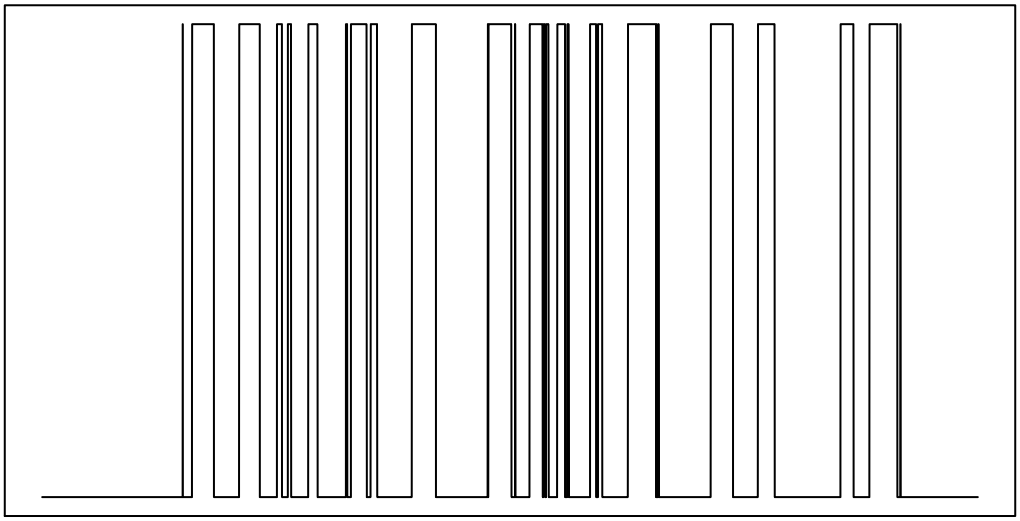

```
plot(event,type='l',cex=.1,ylab='',yaxt='none',xlab='')
```

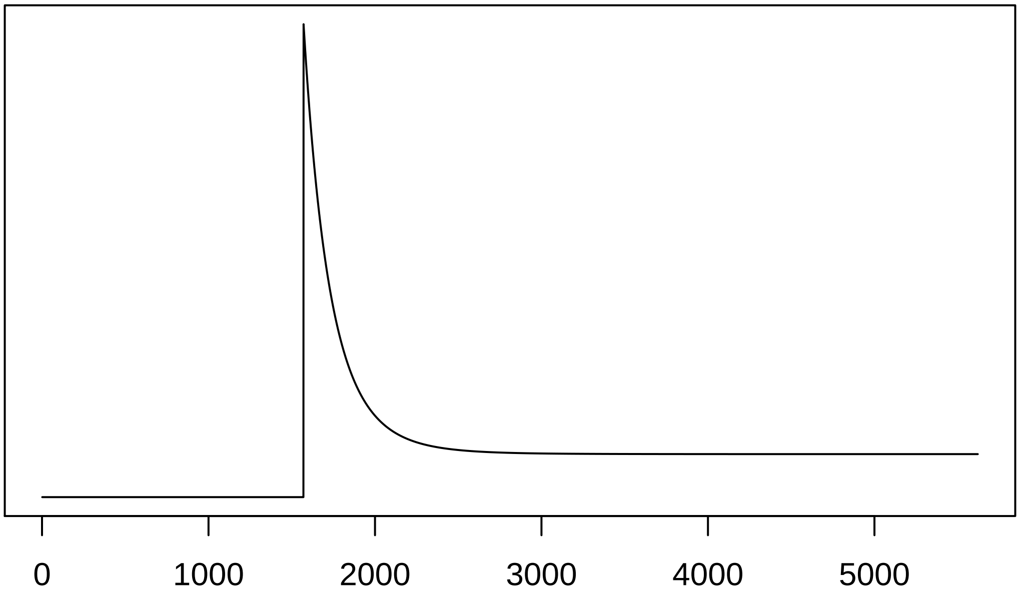


```
plot(twitter_data$total,type='l',col='gray',cex=.1,ylab='',yaxt='none',xlab='',xaxt='none') # the actua
```

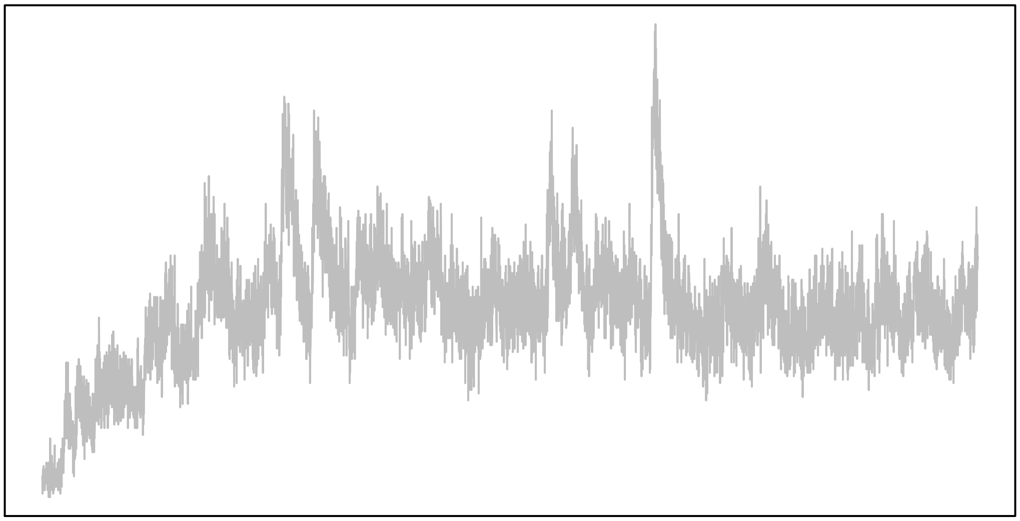

```
for (i in 1:dim(ps)[2]) {
    if (i==1) plot(ps[,i],type='l',col='gray',cex=.1,xlab='',ylab='',yaxt='none')
    else {
        points(ps[,i],type='l',col='gray',cex=.1)
    }
}
```

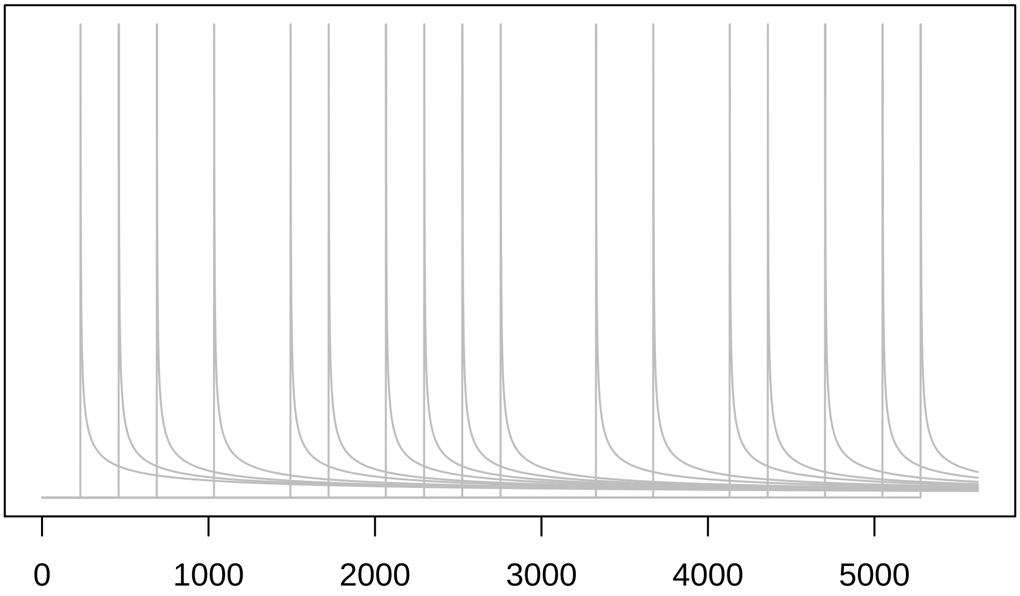


Time to fit our simple model.

```
dv = (twitter_data$total-mean(twitter_data$total))/twitter_data$total
qtime_std = scale(qtime)
lmo_decay = lm(dv~qtime_std)
summary(lmo_decay)
```

```
##
## Call:
## lm(formula = dv ~ qtime_std)
```

```
##
## Residuals:
##     Min      1Q  Median      3Q     Max
## -16.301  -0.243   0.015   0.419   1.373
##
## Coefficients:
##             Estimate Std. Error t value Pr(>|t|)
## (Intercept)  -0.2066     0.0122   -17.0   <2e-16 ***
## qtime_std1    0.3463     0.0122    28.5   <2e-16 ***
## qtime_std2   -0.4630     0.0122   -38.1   <2e-16 ***
## ---
## Signif. codes:  0 '***' 0.001 '**' 0.01 '*' 0.05 '.' 0.1 ' ' 1
##
## Residual standard error: 0.911 on 5618 degrees of freedom
## Multiple R-squared:  0.287,  Adjusted R-squared:  0.287
## F-statistic: 1.13e+03 on 2 and 5618 DF,  p-value: <2e-16
```

```
lmo_decay_lasthalf = lm(dv[length(dv)/2:length(dv)]~qtime_std[length(dv)/2:length(dv),2])
summary(lmo_decay_lasthalf)
```

```
##
## Call:
## lm(formula = dv[length(dv)/2:length(dv)] ~ qtime_std[length(dv)/2:length(dv),
##     2])
##
## Residuals:
##     Min      1Q  Median      3Q     Max
## -12.161   0.917   0.925   0.925   4.551
##
## Coefficients:
##                                         Estimate Std. Error t value Pr(>|t|)
## (Intercept)                                1.050      0.733    1.43     0.15
## qtime_std[length(dv)/2:length(dv), 2]   -3.283      0.330   -9.95   <2e-16
##
## (Intercept)
## qtime_std[length(dv)/2:length(dv), 2] ***
## ---
## Signif. codes:  0 '***' 0.001 '**' 0.01 '*' 0.05 '.' 0.1 ' ' 1
##
## Residual standard error: 2.48 on 5618 degrees of freedom
## Multiple R-squared:  0.0173, Adjusted R-squared:  0.0171
## F-statistic:   99 on 1 and 5618 DF,  p-value: <2e-16
```

```
lmo_all = lm(twitter_data$total~qtime*whoix1C*whoix2C*interruptC+ps) # ps includes many salient event p
lmo_no_ps = lm(twitter_data$total~qtime*whoix1C*whoix2C*interruptC)
lmo_no_interrupt = lm(twitter_data$total~qtime*whoix1C*whoix2C+ps)
lmo_no_whois = lm(twitter_data$total~qtime*interruptC+ps)
lmo_no_qtime = lm(twitter_data$total~whoix1C*whoix2C*interruptC+ps)

anova(lmo_all,lmo_no_ps)
```

```
## Analysis of Variance Table
##
```

```
## Model 1: twitter_data$total ~ qtime * whoix1C * whoix2C * interruptC +
##     ps
## Model 2: twitter_data$total ~ qtime * whoix1C * whoix2C * interruptC
##   Res.Df    RSS  Df Sum of Sq    F Pr(>F)
## 1   5586 772941
## 2   5603 962274 -17   -189333 80.5 <2e-16 ***
## ---
## Signif. codes:  0 '***' 0.001 '**' 0.01 '*' 0.05 '.' 0.1 ' ' 1
```

```
anova(lmo_all,lmo_no_interrupt)
```

```
## Analysis of Variance Table
##
## Model 1: twitter_data$total ~ qtime * whoix1C * whoix2C * interruptC +
##     ps
## Model 2: twitter_data$total ~ qtime * whoix1C * whoix2C + ps
##   Res.Df    RSS Df Sum of Sq    F Pr(>F)
## 1   5586 772941
## 2   5595 805760 -9    -32818 26.4 <2e-16 ***
## ---
## Signif. codes:  0 '***' 0.001 '**' 0.01 '*' 0.05 '.' 0.1 ' ' 1
```

```
anova(lmo_all,lmo_no_whois)
```

```
## Analysis of Variance Table
##
## Model 1: twitter_data$total ~ qtime * whoix1C * whoix2C * interruptC +
##     ps
## Model 2: twitter_data$total ~ qtime * interruptC + ps
##   Res.Df    RSS  Df Sum of Sq    F Pr(>F)
## 1   5586 772941
## 2   5598 864140 -12    -91199 54.9 <2e-16 ***
## ---
## Signif. codes:  0 '***' 0.001 '**' 0.01 '*' 0.05 '.' 0.1 ' ' 1
```

```
anova(lmo_all,lmo_no_qtime)
```

```
## Analysis of Variance Table
##
## Model 1: twitter_data$total ~ qtime * whoix1C * whoix2C * interruptC +
##     ps
## Model 2: twitter_data$total ~ whoix1C * whoix2C * interruptC + ps
##   Res.Df     RSS  Df Sum of Sq   F Pr(>F)
## 1   5586  772941
## 2   5598 1102253 -12   -329311 198 <2e-16 ***
## ---
## Signif. codes:  0 '***' 0.001 '**' 0.01 '*' 0.05 '.' 0.1 ' ' 1
```

```
summary(lmo_all)$r.squared - summary(lmo_no_ps)$r.squared
```

```
## [1] 0.1146
```

```r
summary(lmo_all)$r.squared - summary(lmo_no_qtime)$r.squared
```

```
## [1] 0.1993
```

```r
summary(lmo_all)$r.squared - summary(lmo_no_whois)$r.squared
```

```
## [1] 0.0552
```

```r
summary(lmo_all)$r.squared - summary(lmo_no_interrupt)$r.squared
```

```
## [1] 0.01986
```

```r
debate1model = lmo_no_ps
save(debate1model,file="debate1model.RData")

# let's make sure AR1 does not make all this go away...
#rg = c(2:length(twitter_data$total))
#lmo_all = lm(twitter_data$total[rg]~qtime[rg,]*whoix1C[rg]*whoix2C[rg]*interruptC[rg]+ps[rg]+twitter_d
#lmo_no_ps = lm(twitter_data$total[rg]~qtime[rg,]*whoix1C[rg]*whoix2C[rg]*interruptC[rg]+twitter_data$t
#lmo_no_interrupt = lm(twitter_data$total[rg]~qtime[rg,]*whoix1C[rg]*whoix2C[rg]+ps[rg]+twitter_data$to
#lmo_no_whois = lm(twitter_data$total[rg]~qtime[rg,]*interruptC[rg]+ps[rg]+twitter_data$total[rg-1])
#lmo_no_qtime = lm(twitter_data$total[rg]~whoix1C[rg]*whoix2C[rg]*interruptC[rg]+ps[rg]+twitter_data$to

# the only model that appears to be substantially weakened is the pulse model; interruptions even still
#anova(lmo_all,lmo_no_ps)
#anova(lmo_all,lmo_no_interrupt)
#anova(lmo_all,lmo_no_whois)
#anova(lmo_all,lmo_no_qtime)
```

The above shows that each variable (even interruptions) appears to contribute significantly to the model. Below we show that the fit, overall, is quite good, over r^2 > 0.4 in all three debates.

```r
preds = fitted(lmo_all)
plot(preds,twitter_data$total,type='p',cex=.2,col='gray',xlab='',ylab='',xaxt='none',yaxt='none',xlim=c
axis(1, at = seq(0, 120, by = 20), las=1);axis(2, at = seq(0, 120, by = 20), las=1)
abline(lm(twitter_data$total~preds))
```

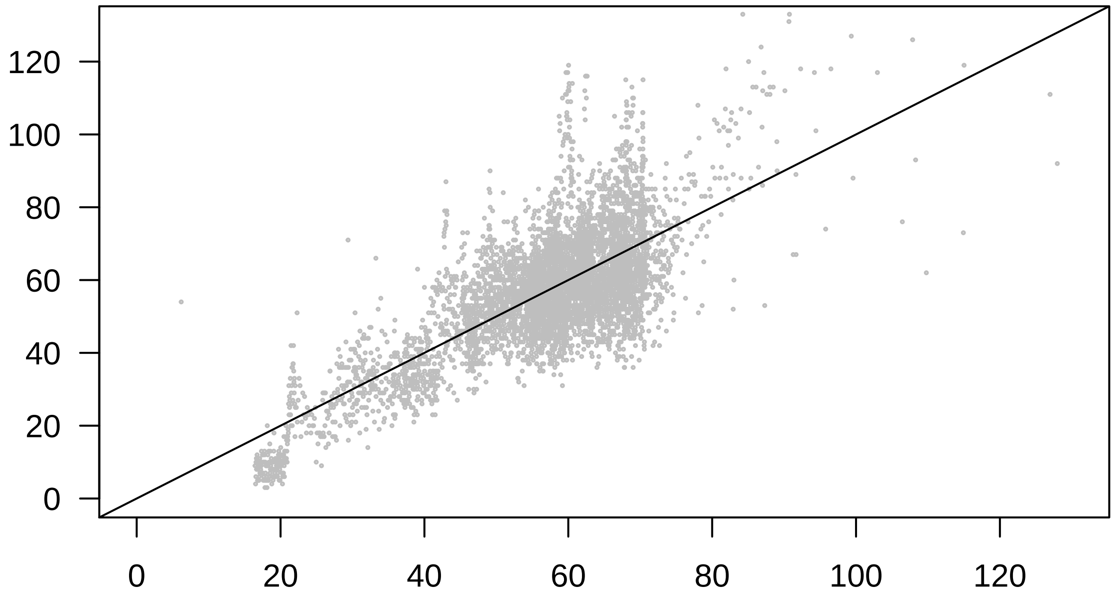


```
#### notes ####

# debate 1 starts in transcripts: 9:01:44
# debate 2 starts in transcripts: 9:01:49
# debate 3 starts in transcripts: 9:01:52
# (timed using stopwatch + CSPAN clock, onset of speech)

# (from riccardo looking into the video / transcript:)
# big bird: 26:11
# binders full of women 38:44
# [horses and ]bayonets 42:03

# (for time codes:)
#3-Interlocutor (1=Romney, 2=Obama, 3=Moderator, 4=Questioner in debate2)
#6-Interruptions1
#   (1=interruption, 2=interrupting and taking the ground)
#7-Interruptions2
#   (1=Moderator interrupts Romney,
#   2=M interrupts O, 3=O interrupts R, 4=R interrupts O,
#   5=R interrupts M, 6=O interrupts M)
```

# A breakdown of the regression model and some plots (Debate 2)

```
setwd('/Users/rickdale/Dropbox/projects/papers/presidentialdebates_full/final_analysis/markdowns/Figure
token_transcript_mention = c(26*60+11,38*60+44,42*60+3) ### the by-the-second occurrence of the meme eve
transcripts_time_starts = c('Wed Oct  3 21:01:44 2012','Tue Oct 16 21:01:49 2012','Mon Oct 22 21:01:52 

dnum = 2 # debate number

debate = read.table(paste('../data/debate',dnum,'.nort.txt',sep=''),head=TRUE,sep='\t') ### get the twi
colnames(debate) = c('date','total','obama','romney','token')

align_index = which(debate$date==transcripts_time_starts[dnum]) ### let's align the debate twitter data
debate = debate[align_index:nrow(debate),] ### start from where the transcript begins (timelocking)

timecodes = read.table('../data/timecodes.txt',sep='\t',head=FALSE) ### get the transcript event segmen
colnames(timecodes) = c('startt','endt','who','speechturn','dnum','interrupt1','interrupt2','topic')
timecodes = timecodes[timecodes$dnum==dnum,] ### only get the current debate for analysis

speakers = c('Romney','Obama','Moderator','Questioner (Debate 2)')
colors = c('red','blue')

# this function finds peaks in the twitter data, indicating potential "salient events"
# models them simply as a slow decay; varies from 0 to 1
pulses = function(tweets) {
  tweets_sm = supsmu(1:length(tweets),tweets,span=.01);
    diffs = diff(tweets_sm$y,2)
    spliney = spline(1:length(tweets),tweets,n=50)
    diffs = diff(spliney$y,1)
    change_dirs = (diffs[2:length(diffs)]<0 & diffs[1:length(diffs)-1]>0)
    indices = which(change_dirs==T)+1
    tweets_peaks = round(spliney$x[indices])
    ps = c()
    for (p in 1:length(tweets_peaks)) {
    ln = length(tweets)-tweets_peaks[p]
    rg = 1:ln
    tail = (rg)
    tail = tail^(-.5)
    psc = c(0*(1:(tweets_peaks[p])),tail)
    ps = cbind(ps,psc)
    }
    pulses = ps
}
```

Create the time series to be used in the regression model.

```
temp = timecodes
temp$who2 = temp$who # who is talking
temp$startt = round(temp$startt) # take it /second
temp$endt = round(temp$endt)    # take it /second
ixes = c() # indices of alignment between twitter and speech (in seconds)
speechturn = c()
```

```
who = c()
whoix1 = c();whoix2 = c()
interrupt = c()
for (j in 1:nrow(temp)) {
  ixes = c(ixes,temp[j,]$startt:temp[j,]$endt)
  speechturn = c(speechturn,(temp[j,]$startt:temp[j,]$endt)*0+temp[j,]$speechturn)
  who = c(who,(temp[j,]$startt:temp[j,]$endt)*0+temp[j,]$who2)
  whoix1 = c(whoix1,1*(temp[j,]$who2==1)*c(1:length(temp[j,]$startt:temp[j,]$endt))) # romney talking i
  whoix2 = c(whoix2,1*(temp[j,]$who2==2)*c(1:length(temp[j,]$startt:temp[j,]$endt))) # obama talking in
  interrupt = c(interrupt,(temp[j,]$startt:temp[j,]$endt)*0+temp[j,]$interrupt1)
}
# since ixes is /second, and will refer to tweet data, remove any 0 that may be at the beginning
if (ixes[1]==0) {
    ixes=ixes[2:length(ixes)];
    who=who[2:length(who)];
    speechturn=speechturn[2:length(speechturn)]
    whoix1 = whoix1[2:length(whoix1)]
    whoix2 = whoix2[2:length(whoix2)]
    interrupt = interrupt[2:length(interrupt)]
}
qtime = poly(1:length(who),2) # rise/fall of the debate
event = c(0*(1:(token_transcript_mention[1]-1)),(token_transcript_mention[1]/(token_transcript_mention[
twitter_data = debate[ixes,] # just get the indices from the twitter data from time 0 to how much of th
ps = pulses(twitter_data$total)
# let's center
whoix2C=(whoix2-mean(whoix2));whoix1C=(whoix1-mean(whoix1));interruptC=interrupt-mean(interrupt);
```

The following code plots the predictors to gain a clear sense of how they will be capturing the variance of the tweet rate for this debate.

```
#dev.new(width=6, height=3)
plot(-1*qtime[,2],type='l',cex=.1,xlab='',ylab='',xaxt='none',yaxt='none') # quadratic time term ("long
points(qtime[,1],type='l',cex=.1)
```

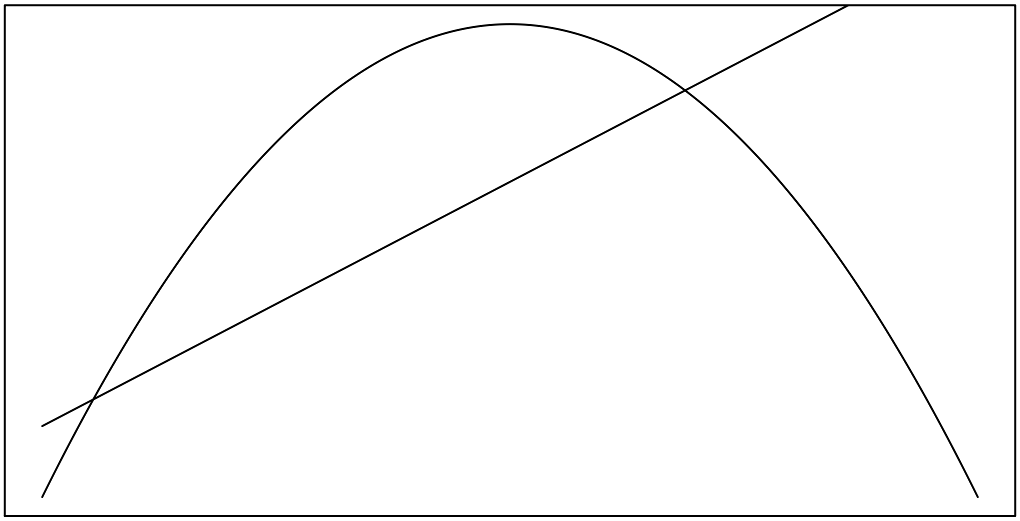

```r
plot(whoix1,type='p',col='red',cex=.1,xlab='',ylab='',xaxt='none',yaxt='none') # romney
```

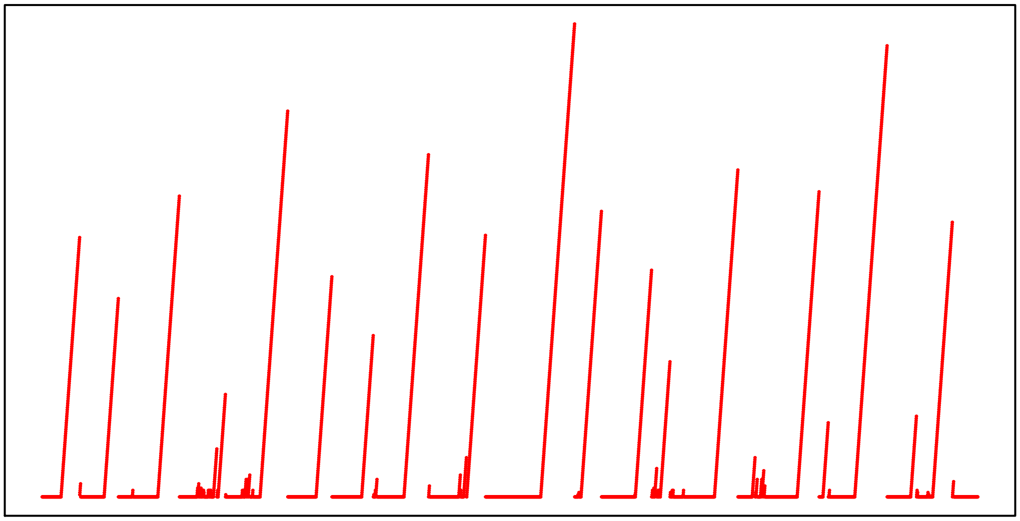

```r
plot(whoix2,type='p',col='blue',cex=.1,xlab='',ylab='',xaxt='none',yaxt='none') # obama
```

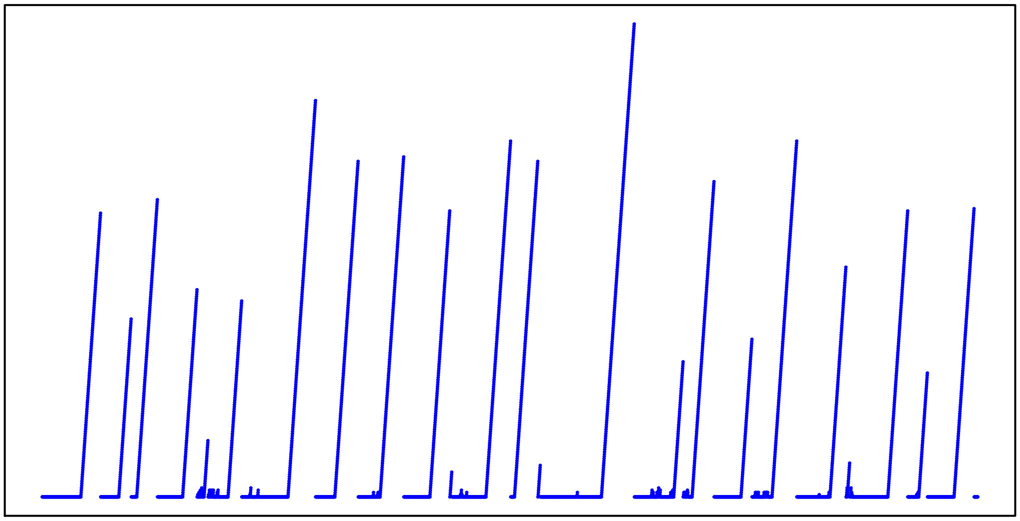

```r
plot(interrupt>0,type='l',cex=.1,ylab='',yaxt='none',xlab='',xaxt='none') # was it an interruption?
```

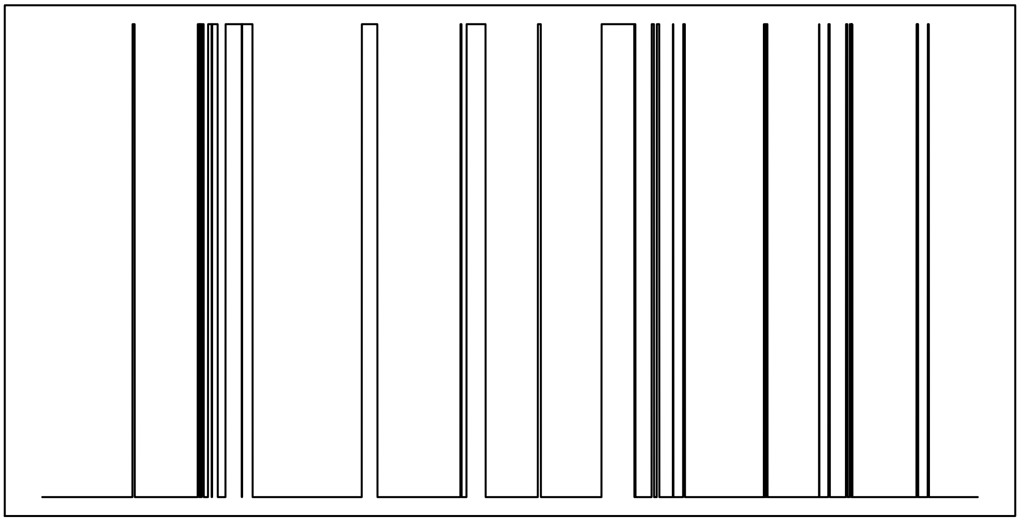

```
plot(event,type='l',cex=.1,ylab='',yaxt='none',xlab='')
```

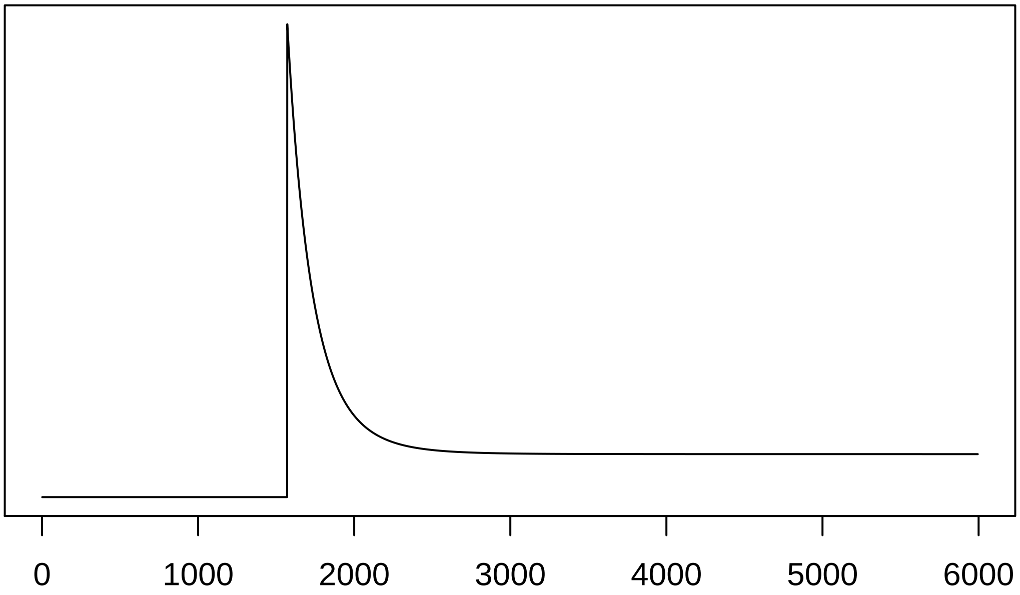


```
plot(twitter_data$total,type='l',col='gray',cex=.1,ylab='',yaxt='none',xlab='',xaxt='none') # the actua
```

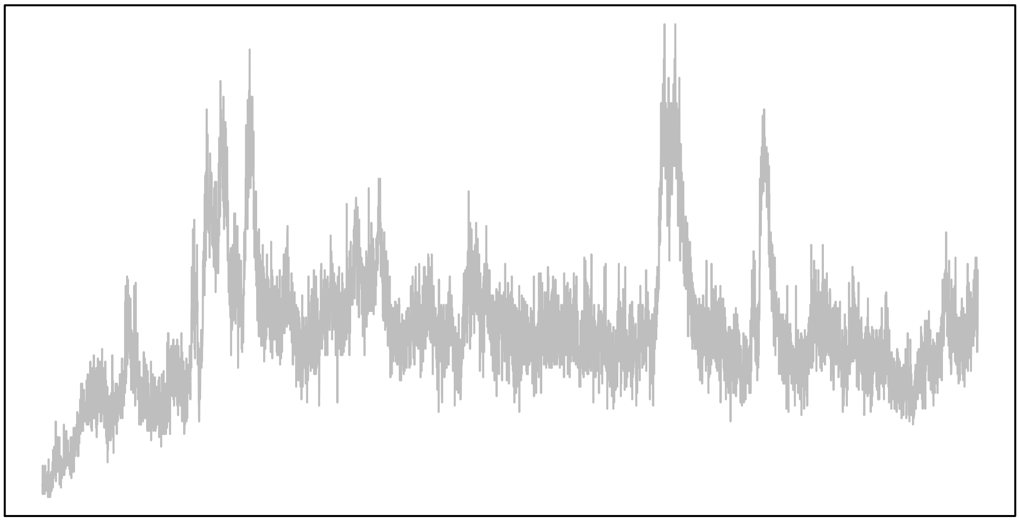

```
for (i in 1:dim(ps)[2]) {
    if (i==1) plot(ps[,i],type='l',col='gray',cex=.1,xlab='',ylab='',yaxt='none')
    else {
        points(ps[,i],type='l',col='gray',cex=.1)
    }
}
```

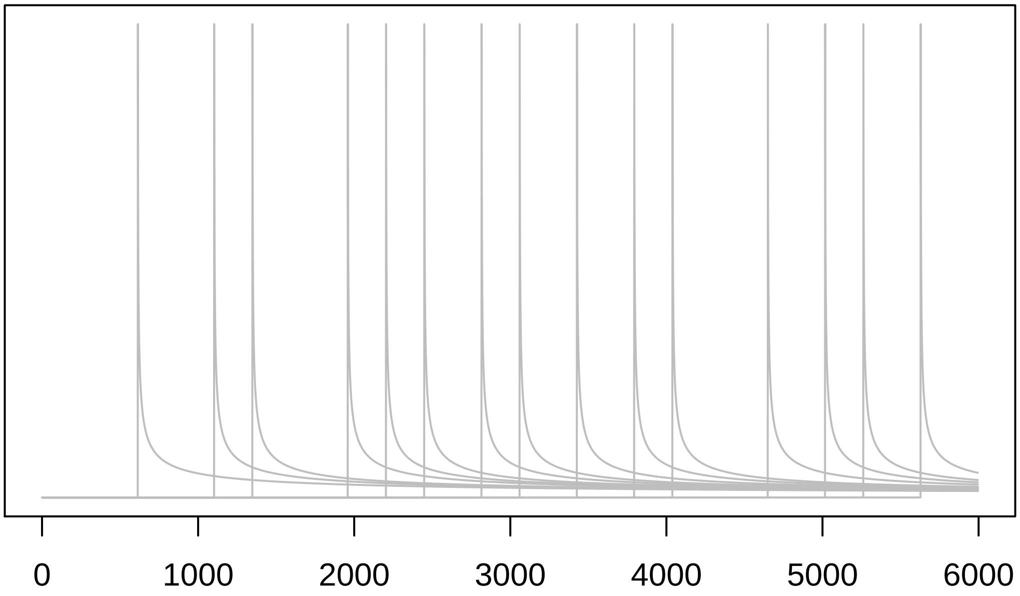


Time to fit our simple model.

```
dv = (twitter_data$total-mean(twitter_data$total))/twitter_data$total
qtime_std = scale(qtime)
lmo_decay = lm(dv~qtime_std)
summary(lmo_decay)
```

```
##
## Call:
## lm(formula = dv ~ qtime_std)
```

```
##
## Residuals:
##     Min      1Q  Median      3Q     Max
## -24.273  -0.293   0.028   0.415   1.610
##
## Coefficients:
##             Estimate Std. Error t value Pr(>|t|)
## (Intercept)  -0.2665     0.0149   -17.9   <2e-16 ***
## qtime_std1    0.3582     0.0149    24.0   <2e-16 ***
## qtime_std2   -0.5256     0.0149   -35.2   <2e-16 ***
## ---
## Signif. codes:  0 '***' 0.001 '**' 0.01 '*' 0.05 '.' 0.1 ' ' 1
##
## Residual standard error: 1.16 on 5992 degrees of freedom
## Multiple R-squared:  0.233,  Adjusted R-squared:  0.232
## F-statistic:  908 on 2 and 5992 DF,  p-value: <2e-16
```

```
lmo_decay_lasthalf = lm(dv[length(dv)/2:length(dv)]~qtime_std[length(dv)/2:length(dv),2])
summary(lmo_decay_lasthalf)
```

```
##
## Call:
## lm(formula = dv[length(dv)/2:length(dv)] ~ qtime_std[length(dv)/2:length(dv),
##     2])
##
## Residuals:
##     Min      1Q  Median      3Q     Max
## -18.135   0.611   0.635   0.635   6.620
##
## Coefficients:
##                                         Estimate Std. Error t value Pr(>|t|)
## (Intercept)                                2.991      0.835    3.58  0.00035
## qtime_std[length(dv)/2:length(dv), 2]   -5.241      0.376  -13.95  < 2e-16
##
## (Intercept)                             ***
## qtime_std[length(dv)/2:length(dv), 2] ***
## ---
## Signif. codes:  0 '***' 0.001 '**' 0.01 '*' 0.05 '.' 0.1 ' ' 1
##
## Residual standard error: 2.82 on 5992 degrees of freedom
## Multiple R-squared:  0.0314, Adjusted R-squared:  0.0313
## F-statistic:  195 on 1 and 5992 DF,  p-value: <2e-16
```

```
lmo_all = lm(twitter_data$total~qtime*whoix1C*whoix2C*interruptC+ps) # ps includes many salient event p
lmo_no_ps = lm(twitter_data$total~qtime*whoix1C*whoix2C*interruptC)
lmo_no_interrupt = lm(twitter_data$total~qtime*whoix1C*whoix2C+ps)
lmo_no_whois = lm(twitter_data$total~qtime*interruptC+ps)
lmo_no_qtime = lm(twitter_data$total~whoix1C*whoix2C*interruptC+ps)

anova(lmo_all,lmo_no_ps)
```

```
## Analysis of Variance Table
##
```

```
## Model 1: twitter_data$total ~ qtime * whoix1C * whoix2C * interruptC +
##     ps
## Model 2: twitter_data$total ~ qtime * whoix1C * whoix2C * interruptC
##   Res.Df     RSS  Df Sum of Sq    F Pr(>F)
## 1   5962 1418693
## 2   5977 1688651 -15   -269958 75.6 <2e-16 ***
## ---
## Signif. codes:  0 '***' 0.001 '**' 0.01 '*' 0.05 '.' 0.1 ' ' 1
```

```r
anova(lmo_all,lmo_no_interrupt)
```

```
## Analysis of Variance Table
##
## Model 1: twitter_data$total ~ qtime * whoix1C * whoix2C * interruptC +
##     ps
## Model 2: twitter_data$total ~ qtime * whoix1C * whoix2C + ps
##   Res.Df     RSS Df Sum of Sq    F Pr(>F)
## 1   5962 1418693
## 2   5971 1525602 -9   -106909 49.9 <2e-16 ***
## ---
## Signif. codes:  0 '***' 0.001 '**' 0.01 '*' 0.05 '.' 0.1 ' ' 1
```

```r
anova(lmo_all,lmo_no_whois)
```

```
## Analysis of Variance Table
##
## Model 1: twitter_data$total ~ qtime * whoix1C * whoix2C * interruptC +
##     ps
## Model 2: twitter_data$total ~ qtime * interruptC + ps
##   Res.Df     RSS  Df Sum of Sq  F Pr(>F)
## 1   5962 1418693
## 2   5974 1512807 -12    -94115 33 <2e-16 ***
## ---
## Signif. codes:  0 '***' 0.001 '**' 0.01 '*' 0.05 '.' 0.1 ' ' 1
```

```r
anova(lmo_all,lmo_no_qtime)
```

```
## Analysis of Variance Table
##
## Model 1: twitter_data$total ~ qtime * whoix1C * whoix2C * interruptC +
##     ps
## Model 2: twitter_data$total ~ whoix1C * whoix2C * interruptC + ps
##   Res.Df     RSS  Df Sum of Sq   F Pr(>F)
## 1   5962 1418693
## 2   5974 1720109 -12   -301416 106 <2e-16 ***
## ---
## Signif. codes:  0 '***' 0.001 '**' 0.01 '*' 0.05 '.' 0.1 ' ' 1
```

```r
summary(lmo_all)$r.squared - summary(lmo_no_ps)$r.squared
```

```
## [1] 0.1056
```

```
summary(lmo_all)$r.squared - summary(lmo_no_qtime)$r.squared
```

```
## [1] 0.118
```

```
summary(lmo_all)$r.squared - summary(lmo_no_whois)$r.squared
```

```
## [1] 0.03683
```

```
summary(lmo_all)$r.squared - summary(lmo_no_interrupt)$r.squared
```

```
## [1] 0.04184
```

```
load("debate1model.RData")
across_debate_preds = predict.lm(debate1model,data.frame(qtime,whoix1C,whoix2C,interruptC))
```

```
## Warning: prediction from a rank-deficient fit may be misleading
```

```
cor.test(across_debate_preds,twitter_data$total)
```

```
##
##  Pearson's product-moment correlation
##
## data:  across_debate_preds and twitter_data$total
## t = 35.03, df = 5993, p-value < 2.2e-16
## alternative hypothesis: true correlation is not equal to 0
## 95 percent confidence interval:
##  0.3911 0.4331
## sample estimates:
##    cor
## 0.4123
```

The above shows that each variable (even interruptions) appears to contribute significantly to the model. Below we show that the fit, overall, is quite good, over r^2 > 0.4 in all three debates.

```
preds = fitted(lmo_all)
plot(preds,twitter_data$total,type='p',cex=.2,col='gray',xlab='',ylab='',xaxt='none',yaxt='none',xlim=c
axis(1, at = seq(0, 120, by = 20), las=1);axis(2, at = seq(0, 120, by = 20), las=1)
abline(lm(twitter_data$total~preds))
```

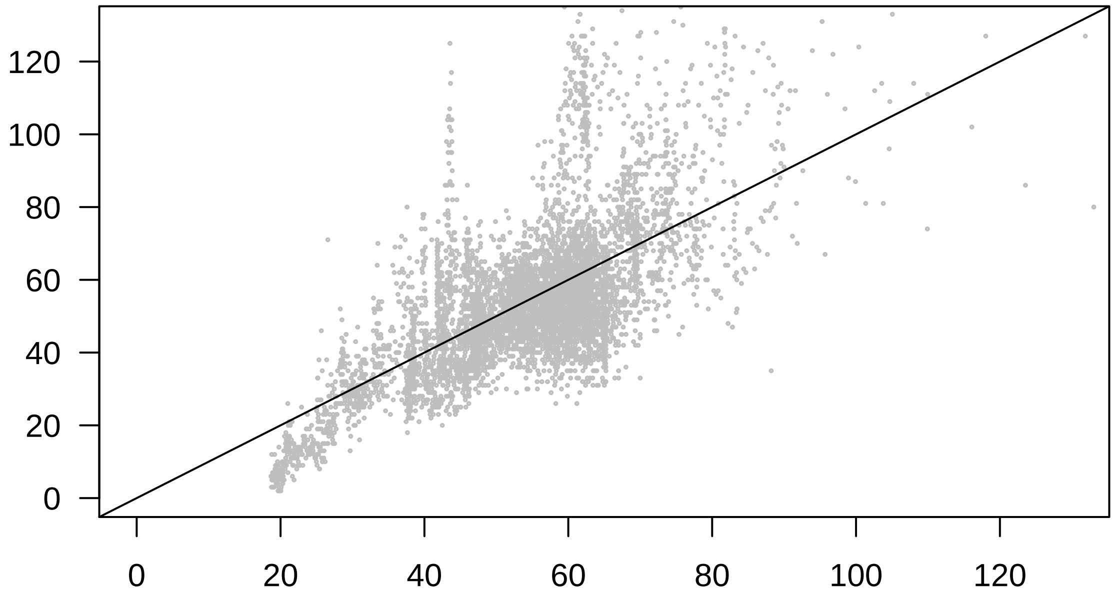


```
#### notes ####

# debate 1 starts in transcripts: 9:01:44
# debate 2 starts in transcripts: 9:01:49
# debate 3 starts in transcripts: 9:01:52
# (timed using stopwatch + CSPAN clock, onset of speech)

# (from riccardo looking into the video / transcript:)
# big bird: 26:11
# binders full of women 38:44
# [horses and ]bayonets 42:03

# (for time codes:)
#3-Interlocutor (1=Romney, 2=Obama, 3=Moderator, 4=Questioner in debate2)
#6-Interruptions1
#   (1=interruption, 2=interrupting and taking the ground)
#7-Interruptions2
#   (1=Moderator interrupts Romney,
#   2=M interrupts O, 3=O interrupts R, 4=R interrupts O,
#   5=R interrupts M, 6=O interrupts M)
```

# A breakdown of the regression model and some plots (Debate 3)

```
setwd('/Users/rickdale/Dropbox/projects/papers/presidentialdebates_full/final_analysis/markdowns/Figure
token_transcript_mention = c(26*60+11,38*60+44,42*60+3) ### the by-the-second occurrence of the meme eve
transcripts_time_starts = c('Wed Oct  3 21:01:44 2012','Tue Oct 16 21:01:49 2012','Mon Oct 22 21:01:52 

dnum = 3 # debate number

debate = read.table(paste('../data/debate',dnum,'.nort.txt',sep=''),head=TRUE,sep='\t') ### get the twi
colnames(debate) = c('date','total','obama','romney','token')

align_index = which(debate$date==transcripts_time_starts[dnum]) ### let's align the debate twitter data
debate = debate[align_index:nrow(debate),] ### start from where the transcript begins (timelocking)

timecodes = read.table('../data/timecodes.txt',sep='\t',head=FALSE) ### get the transcript event segmen
colnames(timecodes) = c('startt','endt','who','speechturn','dnum','interrupt1','interrupt2','topic')
timecodes = timecodes[timecodes$dnum==dnum,] ### only get the current debate for analysis

speakers = c('Romney','Obama','Moderator','Questioner (Debate 2)')
colors = c('red','blue')

# this function finds peaks in the twitter data, indicating potential "salient events"
# models them simply as a slow decay; varies from 0 to 1
pulses = function(tweets) {
  tweets_sm = supsmu(1:length(tweets),tweets,span=.01);
    diffs = diff(tweets_sm$y,2)
    spliney = spline(1:length(tweets),tweets,n=50)
    diffs = diff(spliney$y,1)
    change_dirs = (diffs[2:length(diffs)]<0 & diffs[1:length(diffs)-1]>0)
    indices = which(change_dirs==T)+1
    tweets_peaks = round(spliney$x[indices])
    ps = c()
    for (p in 1:length(tweets_peaks)) {
    ln = length(tweets)-tweets_peaks[p]
    rg = 1:ln
    tail = (rg)
    tail = tail^(-.5)
    psc = c(0*(1:(tweets_peaks[p])),tail)
    ps = cbind(ps,psc)
    }
    pulses = ps
}
```

Create the time series to be used in the regression model.

```
temp = timecodes
temp$who2 = temp$who # who is talking
temp$startt = round(temp$startt) # take it /second
temp$endt = round(temp$endt)    # take it /second
ixes = c() # indices of alignment between twitter and speech (in seconds)
speechturn = c()
```

```
who = c()
whoix1 = c();whoix2 = c()
interrupt = c()
for (j in 1:nrow(temp)) {
  ixes = c(ixes,temp[j,]$startt:temp[j,]$endt)
  speechturn = c(speechturn,(temp[j,]$startt:temp[j,]$endt)*0+temp[j,]$speechturn)
  who = c(who,(temp[j,]$startt:temp[j,]$endt)*0+temp[j,]$who2)
  whoix1 = c(whoix1,1*(temp[j,]$who2==1)*c(1:length(temp[j,]$startt:temp[j,]$endt))) # romney talking i
  whoix2 = c(whoix2,1*(temp[j,]$who2==2)*c(1:length(temp[j,]$startt:temp[j,]$endt))) # obama talking in
  interrupt = c(interrupt,(temp[j,]$startt:temp[j,]$endt)*0+temp[j,]$interrupt1)
}
# since ixes is /second, and will refer to tweet data, remove any 0 that may be at the beginning
if (ixes[1]==0) {
    ixes=ixes[2:length(ixes)];
    who=who[2:length(who)];
    speechturn=speechturn[2:length(speechturn)]
    whoix1 = whoix1[2:length(whoix1)]
    whoix2 = whoix2[2:length(whoix2)]
    interrupt = interrupt[2:length(interrupt)]
}
qtime = poly(1:length(who),2) # rise/fall of the debate
event = c(0*(1:(token_transcript_mention[1]-1)),(token_transcript_mention[1]/(token_transcript_mention[
twitter_data = debate[ixes,] # just get the indices from the twitter data from time 0 to how much of th
ps = pulses(twitter_data$total)
# let's center
whoix2C=(whoix2-mean(whoix2));whoix1C=(whoix1-mean(whoix1));interruptC=interrupt-mean(interrupt);
```

The following code plots the predictors to gain a clear sense of how they will be capturing the variance of the tweet rate for this debate.

```
#dev.new(width=6, height=3)
plot(-1*qtime[,2],type='l',cex=.1,xlab='',ylab='',xaxt='none',yaxt='none') # quadratic time term ("long
points(qtime[,1],type='l',cex=.1)
```

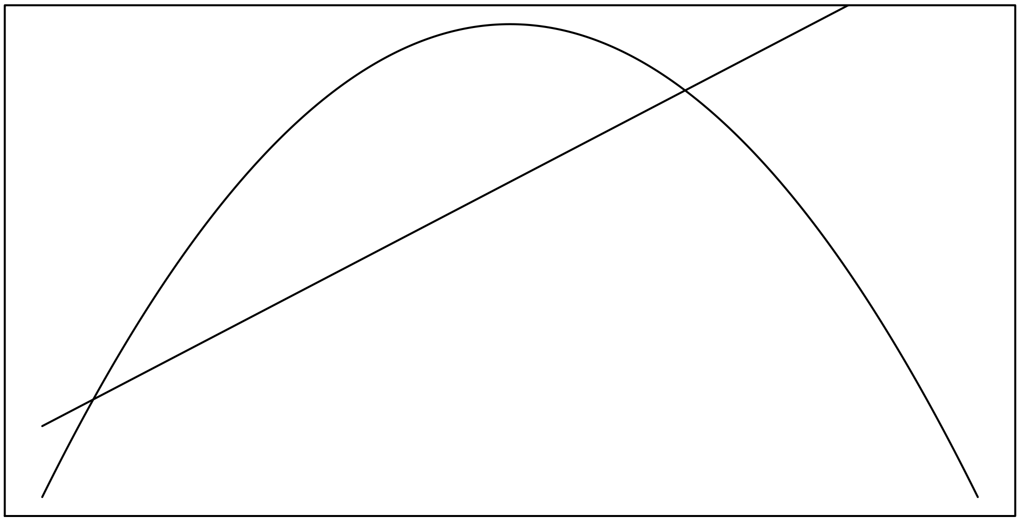

```r
plot(whoix1,type='p',col='red',cex=.1,xlab='',ylab='',xaxt='none',yaxt='none') # romney
```

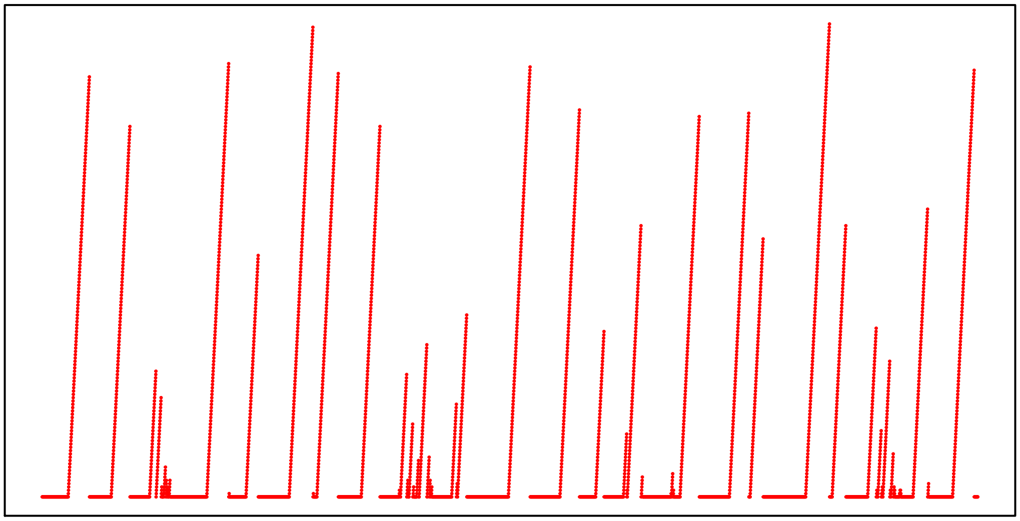

```r
plot(whoix2,type='p',col='blue',cex=.1,xlab='',ylab='',xaxt='none',yaxt='none') # obama
```

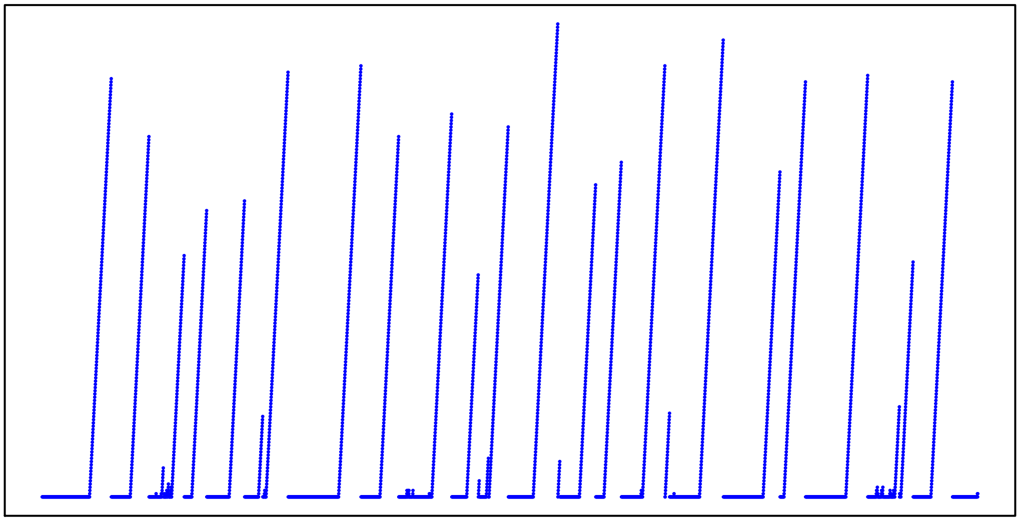

```r
plot(interrupt>0,type='l',cex=.1,ylab='',yaxt='none',xlab='',xaxt='none') # was it an interruption?
```

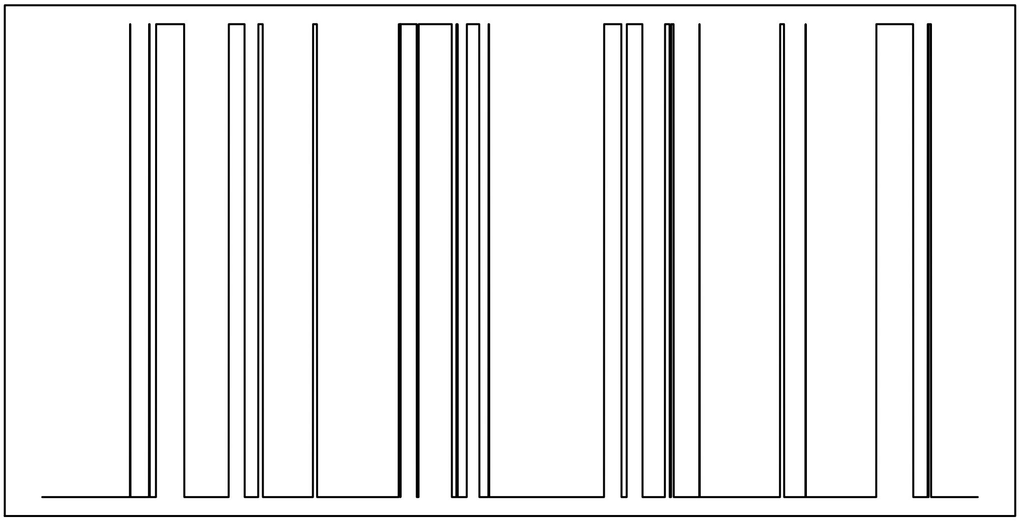

```
plot(event,type='l',cex=.1,ylab='',yaxt='none',xlab='')
```

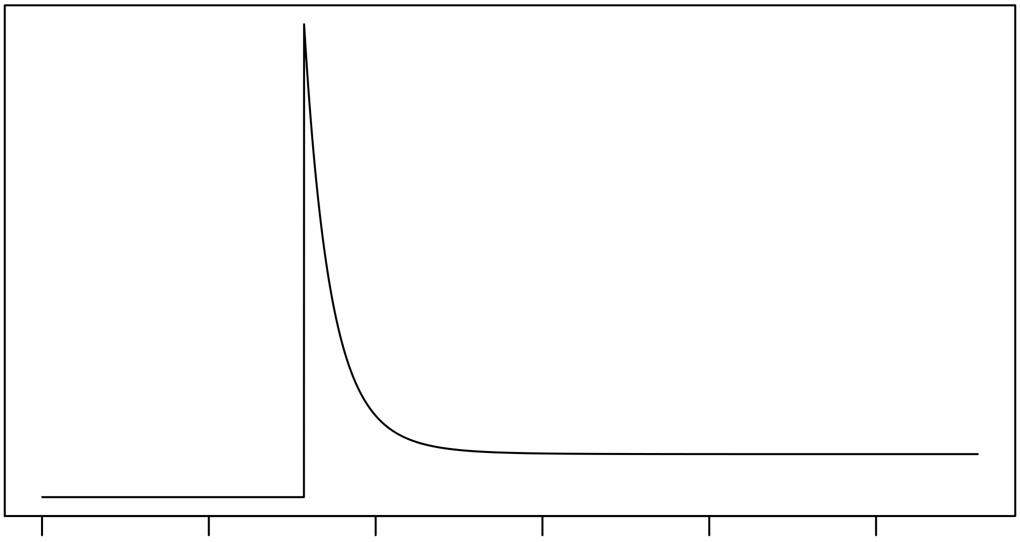


```
plot(twitter_data$total,type='l',col='gray',cex=.1,ylab='',yaxt='none',xlab='',xaxt='none') # the actua
```

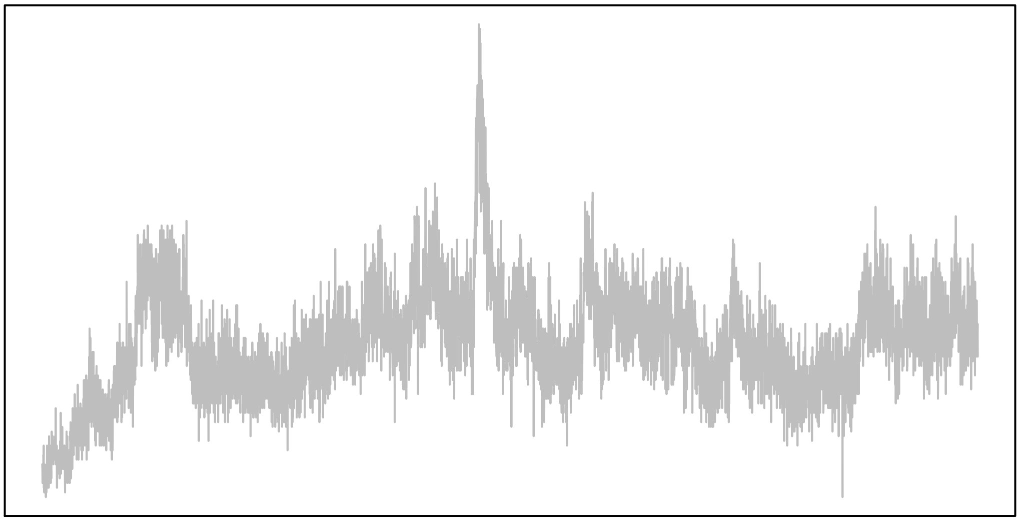

```
for (i in 1:dim(ps)[2]) {
    if (i==1) plot(ps[,i],type='l',col='gray',cex=.1,xlab='',ylab='',yaxt='none')
    else {
        points(ps[,i],type='l',col='gray',cex=.1)
    }
}
```

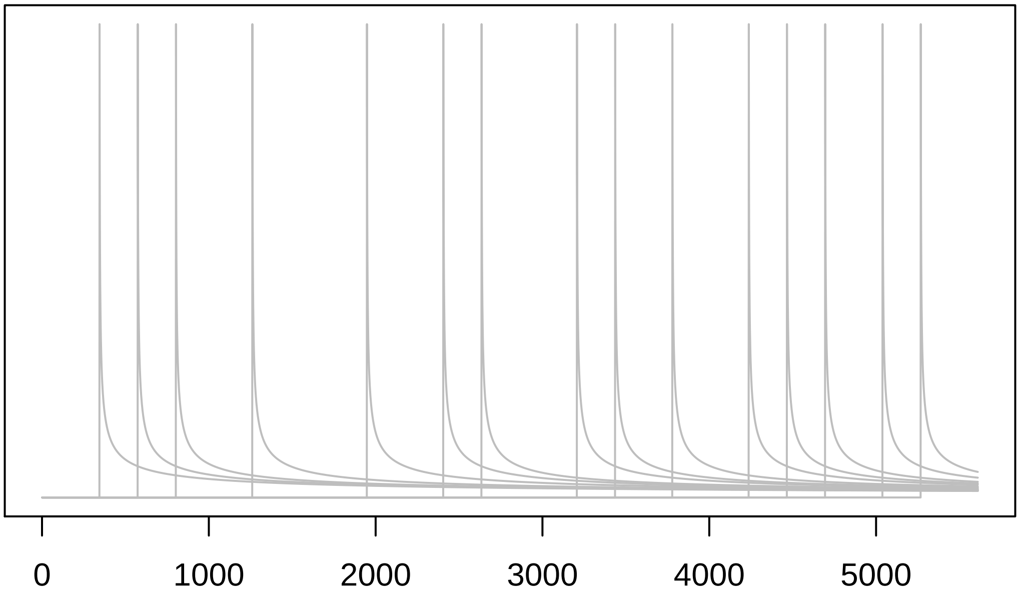


Time to fit our simple model.

```
dv = (twitter_data$total-mean(twitter_data$total))/twitter_data$total
qtime_std = scale(qtime)
lmo_decay = lm(dv~qtime_std)
summary(lmo_decay)
```

```
##
## Call:
## lm(formula = dv ~ qtime_std)
```

```
##
## Residuals:
##    Min     1Q Median     3Q    Max
## -32.70  -0.20   0.06   0.33   1.35
##
## Coefficients:
##             Estimate Std. Error t value Pr(>|t|)
## (Intercept)  -0.2111     0.0133   -15.8   <2e-16 ***
## qtime_std1    0.2781     0.0133    20.9   <2e-16 ***
## qtime_std2   -0.3463     0.0133   -26.0   <2e-16 ***
## ---
## Signif. codes:  0 '***' 0.001 '**' 0.01 '*' 0.05 '.' 0.1 ' ' 1
##
## Residual standard error: 0.999 on 5607 degrees of freedom
## Multiple R-squared:  0.165,  Adjusted R-squared:  0.165
## F-statistic:  555 on 2 and 5607 DF,  p-value: <2e-16
```

```
lmo_decay_lasthalf = lm(dv[length(dv)/2:length(dv)]~qtime_std[length(dv)/2:length(dv),2])
summary(lmo_decay_lasthalf)
```

```
##
## Call:
## lm(formula = dv[length(dv)/2:length(dv)] ~ qtime_std[length(dv)/2:length(dv),
##     2])
##
## Residuals:
##     Min      1Q  Median      3Q     Max
## -28.584   0.427   1.033   1.033   3.296
##
## Coefficients:
##                                        Estimate Std. Error t value Pr(>|t|)
## (Intercept)                              -1.105      0.713   -1.55     0.12
## qtime_std[length(dv)/2:length(dv), 2]   -1.409      0.321   -4.39  1.1e-05
##
## (Intercept)
## qtime_std[length(dv)/2:length(dv), 2] ***
## ---
## Signif. codes:  0 '***' 0.001 '**' 0.01 '*' 0.05 '.' 0.1 ' ' 1
##
## Residual standard error: 2.41 on 5607 degrees of freedom
## Multiple R-squared:  0.00343,    Adjusted R-squared:  0.00325
## F-statistic: 19.3 on 1 and 5607 DF,  p-value: 1.14e-05
```

```
psc = rowSums(ps) - mean(rowSums(ps))

lmo_all = lm(twitter_data$total~qtime*whoix1C*whoix2C*interruptC+psc) # ps includes many salient event
lmo_no_ps = lm(twitter_data$total~qtime*whoix1C*whoix2C*interruptC)
lmo_no_interrupt = lm(twitter_data$total~qtime*whoix1C*whoix2C+ps)
lmo_no_whois = lm(twitter_data$total~qtime*interruptC+ps)
lmo_no_qtime = lm(twitter_data$total~whoix1C*whoix2C*interruptC+ps)

anova(lmo_all,lmo_no_ps)
```

```
## Analysis of Variance Table
##
## Model 1: twitter_data$total ~ qtime * whoix1C * whoix2C * interruptC +
##     psc
## Model 2: twitter_data$total ~ qtime * whoix1C * whoix2C * interruptC
##   Res.Df    RSS Df Sum of Sq    F  Pr(>F)
## 1   5591 539079
## 2   5592 544849 -1     -5770 59.8 1.2e-14 ***
## ---
## Signif. codes:  0 '***' 0.001 '**' 0.01 '*' 0.05 '.' 0.1 ' ' 1
```

```r
anova(lmo_all,lmo_no_interrupt)
```

```
## Analysis of Variance Table
##
## Model 1: twitter_data$total ~ qtime * whoix1C * whoix2C * interruptC +
##     psc
## Model 2: twitter_data$total ~ qtime * whoix1C * whoix2C + ps
##   Res.Df    RSS Df Sum of Sq   F Pr(>F)
## 1   5591 539079
## 2   5586 481371  5     57708 134 <2e-16 ***
## ---
## Signif. codes:  0 '***' 0.001 '**' 0.01 '*' 0.05 '.' 0.1 ' ' 1
```

```r
anova(lmo_all,lmo_no_whois)
```

```
## Analysis of Variance Table
##
## Model 1: twitter_data$total ~ qtime * whoix1C * whoix2C * interruptC +
##     psc
## Model 2: twitter_data$total ~ qtime * interruptC + ps
##   Res.Df    RSS Df Sum of Sq   F Pr(>F)
## 1   5591 539079
## 2   5589 457790  2     81289 496 <2e-16 ***
## ---
## Signif. codes:  0 '***' 0.001 '**' 0.01 '*' 0.05 '.' 0.1 ' ' 1
```

```r
anova(lmo_all,lmo_no_qtime)
```

```
## Analysis of Variance Table
##
## Model 1: twitter_data$total ~ qtime * whoix1C * whoix2C * interruptC +
##     psc
## Model 2: twitter_data$total ~ whoix1C * whoix2C * interruptC + ps
##   Res.Df    RSS Df Sum of Sq   F Pr(>F)
## 1   5591 539079
## 2   5589 503655  2     35424 197 <2e-16 ***
## ---
## Signif. codes:  0 '***' 0.001 '**' 0.01 '*' 0.05 '.' 0.1 ' ' 1
```

```
summary(lmo_all)$r.squared
```

```
## [1] 0.285
```

```
summary(lmo_all)$r.squared - summary(lmo_no_ps)$r.squared
```

```
## [1] 0.007653
```

```
summary(lmo_all)$r.squared - summary(lmo_no_qtime)$r.squared
```

```
## [1] -0.04698
```

```
summary(lmo_all)$r.squared - summary(lmo_no_whois)$r.squared
```

```
## [1] -0.1078
```

```
summary(lmo_all)$r.squared - summary(lmo_no_interrupt)$r.squared
```

```
## [1] -0.07654
```

```
load("debate1model.RData")
across_debate_preds = predict.lm(debate1model,data.frame(qtime,whoix1C,whoix2C,interruptC))
```

```
## Warning: prediction from a rank-deficient fit may be misleading
```

```
cor.test(across_debate_preds,twitter_data$total)
```

```
##
##  Pearson's product-moment correlation
##
## data:  across_debate_preds and twitter_data$total
## t = 25.08, df = 5608, p-value < 2.2e-16
## alternative hypothesis: true correlation is not equal to 0
## 95 percent confidence interval:
##  0.2938 0.3409
## sample estimates:
##    cor
## 0.3175
```

The above shows that each variable (even interruptions) appears to contribute significantly to the model. Below we show that the fit, overall, is quite good, over r^2 > 0.4 in all three debates.

```
preds = fitted(lmo_all)
plot(preds,twitter_data$total,type='p',cex=.2,col='gray',xlab='',ylab='',xaxt='none',yaxt='none',xlim=c
axis(1, at = seq(0, 120, by = 20), las=1);axis(2, at = seq(0, 120, by = 20), las=1)
abline(lm(twitter_data$total~preds))
```

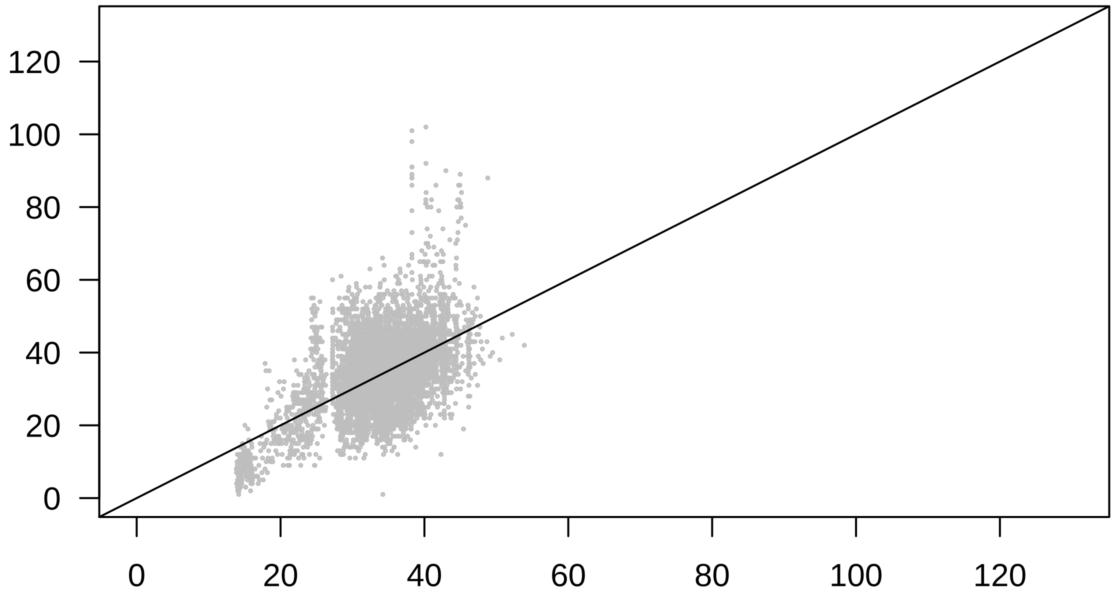


```
#### notes ####

# debate 1 starts in transcripts: 9:01:44
# debate 2 starts in transcripts: 9:01:49
# debate 3 starts in transcripts: 9:01:52
# (timed using stopwatch + CSPAN clock, onset of speech)

# (from riccardo looking into the video / transcript:)
# big bird: 26:11
# binders full of women 38:44
# [horses and ]bayonets 42:03

# (for time codes:)
#3-Interlocutor (1=Romney, 2=Obama, 3=Moderator, 4=Questioner in debate2)
#6-Interruptions1
#   (1=interruption, 2=interrupting and taking the ground)
#7-Interruptions2
#   (1=Moderator interrupts Romney,
#   2=M interrupts O, 3=O interrupts R, 4=R interrupts O,
#   5=R interrupts M, 6=O interrupts M)
```
